# Brain Genomics Superstruct Project initial data release with structural, functional, and behavioral measures

**DOI:** 10.1038/sdata.2015.31

**Published:** 2015-07-07

**Authors:** Avram J. Holmes, Marisa O. Hollinshead, Timothy M. O’Keefe, Victor I. Petrov, Gabriele R. Fariello, Lawrence L. Wald, Bruce Fischl, Bruce R. Rosen, Ross W. Mair, Joshua L. Roffman, Jordan W. Smoller, Randy L. Buckner

**Affiliations:** 1 Center for Brain Science, Harvard University, Cambridge, MA 02138, USA; 2 Department of Psychology, Harvard University, Cambridge, MA 02138, USA; 3 Department of Psychiatry, Massachusetts General Hospital and Harvard Medical School, Boston, MA 02114, USA; 4 Athinoula A. Martinos Center for Biomedical Research, Department of Radiology, Massachusetts General Hospital and Harvard Medical School, Charlestown, MA 02129, USA

**Keywords:** Functional magnetic resonance imaging, Cognitive neuroscience, Brain imaging, Magnetic resonance imaging

## Abstract

The goal of the Brain Genomics Superstruct Project (GSP) is to enable large-scale exploration of the links between brain function, behavior, and ultimately genetic variation. To provide the broader scientific community data to probe these associations, a repository of structural and functional magnetic resonance imaging (MRI) scans linked to genetic information was constructed from a sample of healthy individuals. The initial release, detailed in the present manuscript, encompasses quality screened cross-sectional data from 1,570 participants ages 18 to 35 years who were scanned with MRI and completed demographic and health questionnaires. Personality and cognitive measures were obtained on a subset of participants. Each dataset contains a T1-weighted structural MRI scan and either one (*n*=1,570) or two (*n*=1,139) resting state functional MRI scans. Test-retest reliability datasets are included from 69 participants scanned within six months of their initial visit. For the majority of participants self-report behavioral and cognitive measures are included (*n*=926 and *n*=892 respectively). Analyses of data quality, structure, function, personality, and cognition are presented to demonstrate the dataset’s utility.

## Background & Summary

Recent advances in neuroimaging provide tools to measure structure and map functional networks in the human brain, albeit with limitations inherent to safe, non-invasive approaches^1–3^. The low participant burden of these techniques makes them particularly well suited for large, high-throughput studies. Taking advantage of these innovations, the Brain Genomics Superstruct Project (GSP) was initiated to yield a dataset of structural, functional, behavioral, and genetic information on a large number of clinically normal participants that could be analyzed on its own or combined with other large-scale data collection efforts^4–9^. The dataset is intended to allow exploration of normative properties of brain structure and function, and link individual differences to behavioral phenotypes and genetic origins. The present data descriptor manuscript details the initial release of structural, functional, and behavioral measures.

The approach taken by the GSP is captured in its name—‘superstruct’. ‘Superstruct’ means to erect upon a foundation of another structure. The foundation for the GSP was the large number of ongoing research studies already taking place on matched Siemens 3T Tim Trio scanners across the Boston research community. A rapid structural and functional imaging protocol was developed and shared with the community that could be added onto existing research studies. The brief protocol could also be run during scheduling gaps between other studies. Saliva was collected at the time of scan (for DNA extraction) along with core demographic and health information; a website link was provided for more extensive behavioral phenotyping that included IQ estimates, personality, social and emotional probes, and a series of additional cognitive tasks. By linking existing investigator-initiated studies to a common acquisition protocol, the GSP data aggregation strategy was able to accumulate over 3,000 unique data sets in under 4 years.

Data are documented and shared in standard formats to avoid imposing dependencies on proprietary software and/or processing packages. Beginning with the initial release of data from 1,570 healthy young adults, GSP datasets are selected to encourage investigation in areas of high interest at a scale that would be difficult for individual laboratories to acquire. By compiling and freely distributing these data, we hope to increase the pace of discovery and facilitate future advances in basic and clinical neuroscience.

## Methods

### Utility and limitations of the GSP sample

Features of the GSP acquisition strategy are relevant to understanding the utility and limitations of the dataset. The GSP data collection effort is built on a rapid acquisition protocol being tagged onto existing neuroimaging research studies of healthy control participants. The approach translated new technologies into increased speed of acquisition. The total acquisition time was ~15 min for the basic protocol and ~30 min for more extensive imaging. Thus, the imaging sequences are brief (~2 min for the T1 weighted structural image and ~6 min for each resting-state functional MRI scan). This allowed a very large sample to be acquired quickly and reduced the risk of movement, but also led to attrition, as there were no backup sequences if data quality was compromised. Second, the strategy used a convenience sample from the Boston community that frequently included well-educated individuals with relatively high IQs (many of the college age students are from local colleges with a small fraction coming from Harvard itself). The dispersion of estimated IQ scores is positively shifted relative to the general population. By contrast, many personality traits, such as negative affect, have distributions that would be expected of a clinically-screened population-based sample. Analyses of the GSP data should consider its demographic properties.

### Participants

Between 2008 and 2012 young adults (ages 18 to 35) with normal or corrected-to-normal vision were recruited from the Boston community to participate in the GSP. The 1,570 participants included in the release were selected from a larger database of individuals who participated in the ongoing GSP data collection initiative. Many of the participants were aware of the study through local college recruitment efforts and through studies connected to Harvard University and the Massachusetts General Hospital. Among the participants enrolled as college students, only a minority were recruited directly from students of Harvard University. Participants were only enrolled if they were participating in a study of normal (non-clinical) brain function or serving as a control participant in a case-control study of a clinical population. Participants provided written informed consent in accordance with guidelines established by the Partners Health Care Institutional Review Board and the Harvard University Committee on the Use of Human Subjects in Research (See Supplementary Appendix A for representative study consent forms). Only those individuals who agreed to data sharing are included in the present release. The broader GSP control sample, which has over 3,000 participants, includes individuals over the age of 35 and also several hundred datasets acquired using a 32-channel head coil. The present release represents a subset of the control sample, ages 18–35, acquired uniformly on the same model 12-channel head coil, with data meeting quality control criteria as described below.

Participation in the GSP comprised four components. Participants were asked to: 1) complete a basic set of demographic and health questionnaires just before, or directly after, the scan; 2) undergo a series of structural and functional MRI scans; 3) provide a saliva sample before and after the scan; and 4) complete a web-based battery of behavioral, cognitive, and personality assessments. Demographic and health questionnaires included information concerning the participants’ physical health, past and present history of psychiatric illness, medication usage, and family history of psychiatric illness. Participants were excluded from the present data release if their self-reported health information indicated current/past history of Axis I pathology or neurological disorder, current psychotropic medication usage and/or acute physical illness, or displayed atypical brain anatomy (*n*=218).

Analyses of portions of the demographic, behavioral, and imaging data obtained from participants in this release have been previously reported^10–22^.

### MRI data acquisition

All imaging data were collected on matched 3T Tim Trio scanners (Siemens Healthcare, Erlangen, Germany) at Harvard University and Massachusetts General Hospital using the vendor-supplied 12-channel phased-array head coil. Structural data included a high-resolution (1.2 mm isotropic) multi-echo T1-weighted magnetization-prepared gradient-echo image (multi-echo MPRAGE^23^; Table 1; See Supplementary Appendix B for relevant DICOM header field values). The low participant burden resulting from the use of multi-echo MPRAGE anatomical scans makes this sequence well suited for high-throughput studies. The morphometric features derived through conventional 6-min 1 mm MPRAGE and the 2-min 1.2 mm multi-echo MPRAGE are highly consistent (r^2^>0.9 for most structures)^24^ suggesting that rapid acquisition multi-echo MPRAGE can be used for many purposes in place of longer anatomical scans without degradation of the quantitative morphometric estimates. Rapid acquisition is also beneficial because it lessens the opportunity for within-scan motion.

Functional imaging data were acquired using a gradient-echo echo-planar imaging (EPI) sequence sensitive to blood oxygenation level-dependent (BOLD) contrast (Table 1; See Supplementary Appendix C for relevant DICOM header field values)^25,26^. Whole brain coverage including the entire cerebellum was achieved with slices aligned to the anterior commissure-posterior commissure plane using an automated alignment procedure that ensured consistency among subjects^27^. BOLD runs consisted of 47 interleaved slices (foot—head; 1, 3, 5 … 45, 47, 2, 4, 6 …, 44, 46). One hundred and twenty-four measurements were collected for each BOLD run (TR=3000 msec; 4 initial TRs collected to allow for T1-stablization and 120 valid measurements). During BOLD data collection participants were instructed to remain still, stay awake, and keep their eyes open while blinking normally. Eyes open rest, without the use of a fixation cross-hair, was chosen because of comparability to fixation (in contrast to eyes closed rest) in provisional tests^28^ and critically because it did not require a visual apparatus which was not always available through the base research studies. One or two BOLD runs were acquired per subject (72.6% of sessions included two runs). Software upgrades (B13, B15, B17) occurred over the course of the data collection and reflect the only known difference in acquisition that took place across the 1,570 sessions. The software version is included in the data descriptors to allow it to be co-varied, and the test-retest data include individuals scanned between software versions to quantify effects, if any, of software version.

### Online cognitive and self-report batteries

Following MRI data collection, participants were provided a card with a random de-identified code and two web addresses to conduct an online battery of cognitive, behavioral, and personality assessments spanning a broad range of domains (See Supplementary Appendix D for a list of the phenotypes included in the present release). The behavioral and personality assessments were hosted on a secure internal server and presented through the LimeSurvey user interface (http://www.limesurvey.org/). Cognitive assessments were presented through an internally developed collection of standard cognitive assessments administered using Adobe Flash from Creative Suite 3 (http://www.adobe.com/; see Code availability). Prior work indicates that self-selected participants completing unsupervised online batteries of cognitive and perceptual tasks can provide data consistent with traditionally recruited and/or lab-tested samples^29^. As an additional quality control procedure, participants’ data were excluded if they demonstrated non-compliance during either battery. Participants were considered non-compliant for the behavioral and personality portion if they failed to initiate or did not complete the entire online assessment, failed to answer more than two questions, or admitted to seeking outside assistance during the completion of the battery. Participants’ were considered non-compliant during the online cognitive portion if they committed more than two errors in a simple keyboard response task, made an erroneous response on one or more ‘catch’ trials placed through the session, responded with excessively slow response times (≥2 s), or failed to complete the battery. ‘Catch’ trials consisted of simple trials designed to seamlessly integrate with the administered tasks. They are correctly answerable with minimal effort/attention on the part of the participants and meant to identify gross non-compliance with task instructions. The Profile of Mood States (POMS)^30^ was incorporated into the self-report battery following the start of GSP data collection. Accordingly, this measure is available in a subset of the broader sample (*n*=897).

### Genetics collection

Saliva samples were collected using two Oragene saliva kits (Oragene, DNA Genotek). The initial saliva sample was collected after consenting the participant, immediately prior to the scan. For backup purposes, a second saliva sample was collected immediately following the scan. The genetic data are planned for release in the future but are not included as part of this initial data release.

### Data security

Data from all paper surveys, MRI acquisitions, and test batteries were archived in a custom deployment of the eXtensible Neuroimaging Archive Toolkit (XNAT)^31^. Access was restricted by user authentication and role-based access controls. Each dataset was uploaded via the DICOM protocol to the XNAT system from the MRI scanner console directly, or from an external application such as DicomBrowser (http://hg.xnat.org/dicombrowser). Newly uploaded data were stored in a temporary ‘PreArchive.’ Designated study staff ‘Archived’ datasets into the respective XNAT Project that identified the laboratory that collected the data. Once a dataset was assigned to a Project, those data were only viewable by users assigned read privilege to that Project.

### Quality control

Movement and degraded data quality can confound results^15,32–34^. Images in the GSP were screened for artifacts, acquisition problems, processing errors and excessive motion. Each image was viewed on a per-slice basis along each principal axis. Typical data quality issues included electronic noise resulting in bright lines through multiple slices, motion artifacts appearing as hazy bands across the image, poor head positioning resulting in wraparound artifacts, distortions from dental work, and limited image contrast (*n*=54). BOLD scans with slice-based temporal signal-to-noise ratio (sSNR) less than 100 were excluded from the release dataset (*n*=88; See Technical Validation; measures of rest scan data quality).

BOLD functional runs were automatically processed through the Automated Functional MRI Quality Assessment tool^35^ to derive estimates of slice-based temporal sSNR, number of relative translations in 3D space ≥0.1 mm (micro-movements), and maximum absolute translation in 3D space (mm). The slice-based SNR was calculated as the weighted mean of each slice’s mean intensity over time (weighted by the size of the slice). The number of movements ≥0.1 mm was calculated by determining the root mean square of the rigid body translations and rotations for motion correction using MCFLIRT from the FSL suite^36^. Each series was aligned to the initial TR, after dropping the first four image volumes for signal stabilization purposes. The maximum absolute translation was calculated as the absolute value of the maximum movement observed.

### Post-processing

Imaging data were converted from DICOM to NIfTI-1 format (http://nifti.nimh.nih.gov/) using mri_convert from FreeSurfer v4.5.0 (http://surfer.nmr.mgh.harvard.edu/). The de-identification of the high-resolution anatomical images was completed through the mask_face software^37^, which ‘blurs’ facial anatomy. Facial blurring was selected for data anonymization as traditional skull stripping algorithms may lead to the failure of automated pre-processing pipelines and/or remove anatomical features necessary for the calculation of intracranial volume and cerebrospinal fluid volume^37^.

Users should be aware that face distortion of anatomical data could influence morphometric estimates. These effects are not uniform across the brain and can arise from the template registration procedures and other steps that are embedded within automated processing pipelines. To characterize the effect of face blurring on analyses of brain anatomy, the release data were processed in FreeSurfer 4.5.0 both before and after de-identification. FreeSurfer provides automated algorithms for subcortical volumetric segmentation and the estimation of cortical thickness^38,39^, allowing users to analyze estimated cortical thickness independent of cortical volume. Cortical thickness was calculated as the closest distance from the gray/white boundary to the gray/CSF boundary at each vertex on the tessellated surface^39^. Using the strategy detailed in Buckner *et al.* ^40^, a study-optimized reference template was created from 700 subjects available through the existing dataset.

Subjects whose automated morphometric assessments of head size changed by more than 1.5% as a function of face blurring were excluded from the data release (*n*=16). Analyses of the released data revealed that large morphometric features, such as estimated intracranial volume, were robust to the effects of face blurring (Pearson r=0.99; Supplementary Fig. 1). However, subcortical volumes (e.g., amygdala, r=0.96) and estimated cortical thicknesses (e.g., medial prefrontal cortical thickness, r=0.90) proved more sensitive to the effects of face blurring. To provide pre-blurred estimates of brain structure, the morphometric values for each participant, computed on their respective raw anatomical scans through FreeSurfer 4.5.0, are included in the initial data release. Participants were processed in a fully automated manner, without manual corrections, and the resulting data were visually inspected for errors. Nonetheless, end users of the present dataset and other datasets that use face blurring (e.g., the NIH Human Connectome Project) should be aware that the procedure could induce subtle effects on data processing, especially if standard non-tailored atlas targets are employed.

### Code availability

Study data were archived with the XNAT open-source imaging informatics software platform^31^ (http://www.xnat.org/). Neuroimaging data were analyzed through the use of standard processing pipelines (e.g., http://surfer.nmr.mgh.harvard.edu/). Online survey data were collected through the LimeSurvey user interface (http://www.limesurvey.org/). Online cognitive batteries were administered through an internally developed collection of standard cognitive assessments administered using Adobe Flash from Creative Suite 3 (http://www.adobe.com/). Simple procedural Actionscript configured the responses to be captured for each trial and the order in which the trials were presented to the subject. Recorded responses were sent to a PHP server and stored in a MySQL database. Over the course of the GSP collection data were downloaded using the server's web dashboard. The custom cognitive battery code is hard linked to libraries and images we do not have the authority to distribute. Accordingly, the code used for this specific component is currently not freely available for download.

## Data Records

### Obtaining the dataset

The ‘Brain Genomics Superstruct Project (GSP)’ initial release dataset of structural, functional, and behavioral measures is available for download (http://neuroinformatics.harvard.edu/gsp/). Step-by-step instructions detailing how to access the release dataset are available online in the ‘Request Access’ page (http://neuroinformatics.harvard.edu/gsp/get). Details regarding the format of available data, imaging sequences, download procedures, as well as answers to commonly asked questions and the description of phenotypes included in the present release are provided in the GSP_README_140630.pdf file (updated versions will be named accordingly to reflect release date, e.g., GSP_README_150530.pdf). Users are encouraged to view the accompanying video tutorials. Data are made available through the Harvard Dataverse Network (http://neuroinformatics.harvard.edu/gsp/dataverse, Data Citation 1). The LONI Image Data Archive provides an additional option for data download (Data Citation 2).

Briefly, in Dataverse imaging data are stored in 10 separate tar files, each containing 157 subjects. All 10 of the tar files must be downloaded to obtain the full dataset. In addition, there is a single description comma separated value (.csv) file (GSP_list_140630.csv) in the study ‘Documentation’ section that contains the demographic and phenotype data for all 1,570 unique subjects. Test-retest data are stored separately in a single tar file (GSP_retest_140630.tar) that contains both sessions for each of the 69 test-retest participants. A single description .csv file (GSP_retest_140630.csv) for the 69 test-retest subjects is included as well. Downloading the single test-retest tar file provides all of the data needed for analysis of reliability.

An extended set of phenotypes, listed in italics in Supplementary Appendix D, is available as part of an additional download. Presently this download is available from the LONI Image Data Archive. Given the increased sensitivity of the extended phenotypes, the LONI approval process requires that all users sign and mail, email, or fax a separate GSP Restricted Data Use Terms application to the Harvard Neuroinformatics Research Group. The internal review and approval process is based on the same criteria outlined in the GSP Restricted Data Use Terms application. Access will be provided to (1) Principal Investigators (PIs) of scientific research at a university, a research organization (including commercial entities) or a government agency who is the leader of a laboratory or research team or who is working independently; or (2) to users who provide the name of the PI who is overseeing their research and is approved for access under qualification 1. If a user does not meet either of the above criteria they may be considered qualified based on a track record of scientific publications or on the basis of a written reference from someone who meets qualification 1, verifying that the data will be used only for the purpose of legitimate scientific research. This restricted access procedure is modeled after the successful Washington University—Minnesota Human Connectome Project. Step-by-step instructions detailing how to access the extended release dataset are available online (http://neuroinformatics.harvard.edu/gsp/get). The extended release contains an additional .csv file (GSP_extended_140630.csv).

### Imaging data

The GSP datasets are available in NIfTI-1 file format. Table 1 provides descriptions of the available sequences. Information available in the original DICOM header (e.g., precise slice acquisition timing) is provided in Supplementary Appendices B and C. The structural images are T1-weighted multi-echo MPRAGE images that are collected with 1.2 mm isotropic resolution^23^. The single anatomical image file contained in the release is the root mean square (RMS) average of the four echoes that were acquired. For most analytic purposes, the RMS average can be treated as a standard structural T1-weighted image. The BOLD data acquisitions include four timepoints before T1-stabilization has occurred. These images have increased signal relative to the remaining timepoints and should be discarded for most BOLD series analyses. The initial image, given its contrast and weighting, can be used for registration.

### Phenotypic information

All phenotypic data are stored in .csv files. A description of phenotypes included in the present release is provided in Supplementary Appendix D and also appears in the GSP_README_140630.pdf file. Several phenotypes, listed in italics at the end of the Phenotypes Legend list, are separately available for download as part of an extended data release (http://neuroinformatics.harvard.edu/gsp/get).

## Technical Validation

### Overview

The current release contains data from 1,570 participants (age: 21.5±2.9; female: 57.6%; right handed: 92.3%; years of education: 14.5±1.9; estimated IQ 110.7±6.7). Additional demographic characteristics of the participants are reported in Table 2. Participants were recruited from Boston area universities and colleges, and the surrounding communities. Consistent with this recruitment strategy, approximately 92% of the sample was under the age of 27 at the date of scan (Table 3). As detailed in Supplementary Appendix D, to protect participant identity, select demographic features and details of data collection have been removed or binned.

### Reliability scans

Development of meaningful imaging-based measures and biomarkers requires estimates of phenotype reliability^41^. To support this need, a supplementary dataset (*n*=69) was acquired over the course of the primary collection effort. Data were collected on two independent days separated by less than 6 months (77.2±55.9 days). These data can be used to estimate test-retest reliability for existing morphometric and functional measures as well as the refinement and evaluation of novel methods and coupled with existing open science resources for the assessment of test-retest reliability (e.g., http://fcon_1000.projects.nitrc.org/indi/CoRR/html/)^41^. Many of the test pairs were acquired across two different scanners or across software console versions allowing reliability estimates that truly reflect the main sources of variance across the GSP sample.

As a demonstration of the utility of the reliability scan pairs, the structural images from each independent session were processed through the automated FreeSurfer pipeline separately. Pearson correlations were used to compare the morphometric estimates across the two visits (Table 4). Correlations range from 0.75 for the estimated cortical thickness of the right medial prefrontal cortex to 0.99 for the estimated intracranial volume. The observed patterns of regional variation in reliability could arise from instability in the morphometric pipeline, scan-rescan shifts in head positioning, hydration, or motion, which may disproportionately impact estimates of small structures and cortical thickness^42–45^. These reliability data are provided for analysis in isolation or to be combined with developing open repositories of reliability data^41^.

### Construct validity of anatomic data

**Figure 1:**
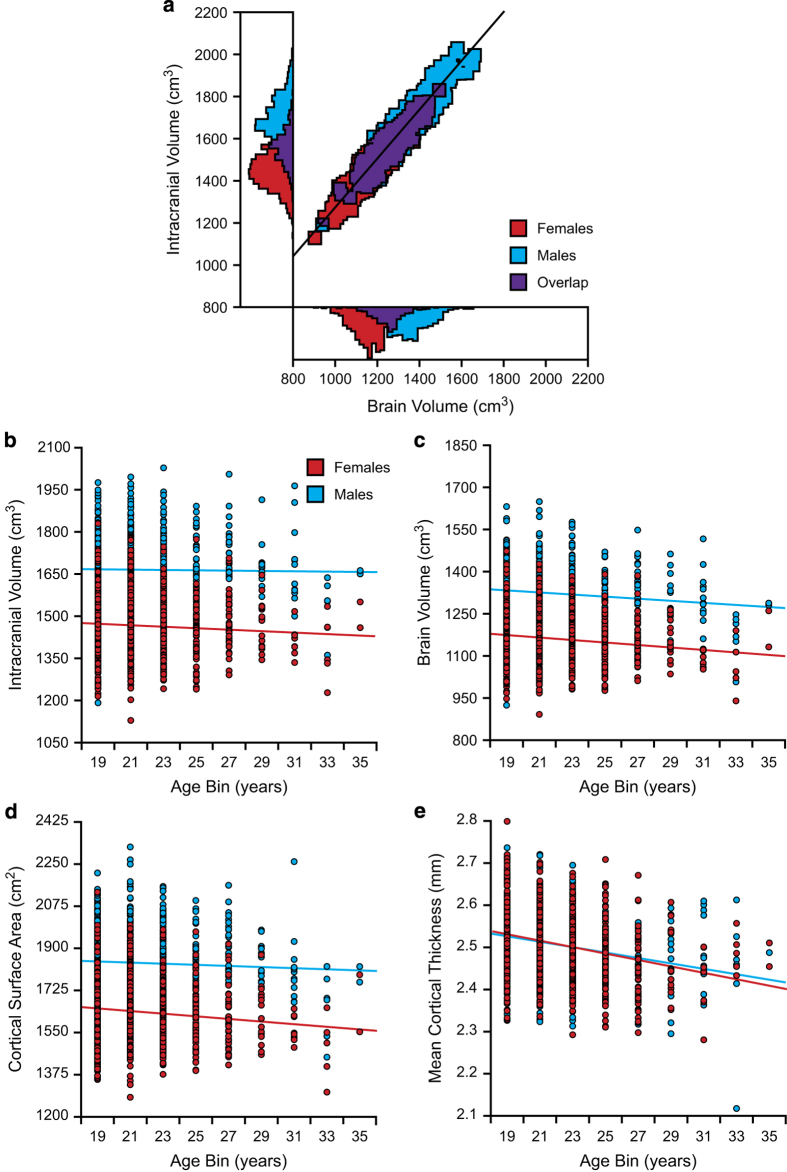
Structural brain volume and morphometric measures. (**a**) A scatter plot of the derived structural MRI estimates from the 1,570 participants included in the present data release reveals expected relations between sex, intracranial volume (ICV), and brain volume. Histograms of both brain volume and ICV are represented on the x and y axes respectively. (**b**–**e**) Scatter plots display the correlations between age (2 year bins) and morphometric estimates of (**b**) ICV (Females r=−0.07; Males r=−0.01), (**c**) brain volume (Females r=−0.14; Males r=−0.11), (**d**) cortical surface area (Females r=−0.12; Males r=−0.05), and (**e**) mean cortical thickness (Females r=−0.28; Males r=−0.26). Note ICV differs by sex but minimally by age reflecting the sex difference in head size that is achieved by adolescence and remains stable. By contrast, cortical thickness is nearly identical between the sexes but decreases progressively with age.

Having established the reliability of the morphometric estimates, anatomical features were analyzed to validate that commonly observed relations are present in the data and of typical magnitude. Estimated intracranial volume (ICV) and total brain volume are plotted in Fig. 1. As expected for a group of young adults where neurodegenerative processes have not begun^46^, ICV is highly correlated with brain volume (r=0.96). Head size differs between men and women^40,47,48^. Consistent with larger head size^40,49,50^, males displayed increased ICV, brain volume, and cortical surface area, relative to females, ranging from 12.4 to 13.8% [Fig. 1a–d; t(1568)=33.95, 32.88, 29.50, respectively; all ps<0.001]. Effective head size normalization should correct this difference. As highlighted in Supplementary Fig. 2, head size normalization accounts for sex differences in regional and whole-brain morphometric analyses^40^. No relations with sex emerged in the raw (uncorrected) data when considering average cortical thickness [t(1568)=0.80, *P*=0.43] consistent with models, and prior data, that suggest thickness increases minimally with head size^49^. Of interest, there was also no sex difference noted for cortical thickness as predicted by early neurodevelopmental models that hypothesize cortical surface area but not thickness differs across normal variability in head size. The dissociation between effects on surface area and thickness is quite dramatic in the contrasting plots of Fig. 1d and Fig. 1e.

ICV is stable across the adult lifespan. In the present data participant age did not associate with estimated ICV (r=−0.01; *P*=0.66). Brain volume (r=−0.08; *P*<0.005) and cortical surface area (r=−0.05; *P*<0.05) displayed modest relations, perhaps reflecting brain volume loss which is thought to be present, but small, in this age range^50^. Even with the compressed age range in the present sample, average cortical thickness was inversely associated with age (r=−0.27; *P*<0.001; Fig. 1e). Taken together, these results demonstrate that age-associated shifts in brain anatomy are evident early in life and that the extent of these effects varies based on the phenotype of interest.

### Functional data quality

**Figure 2:**
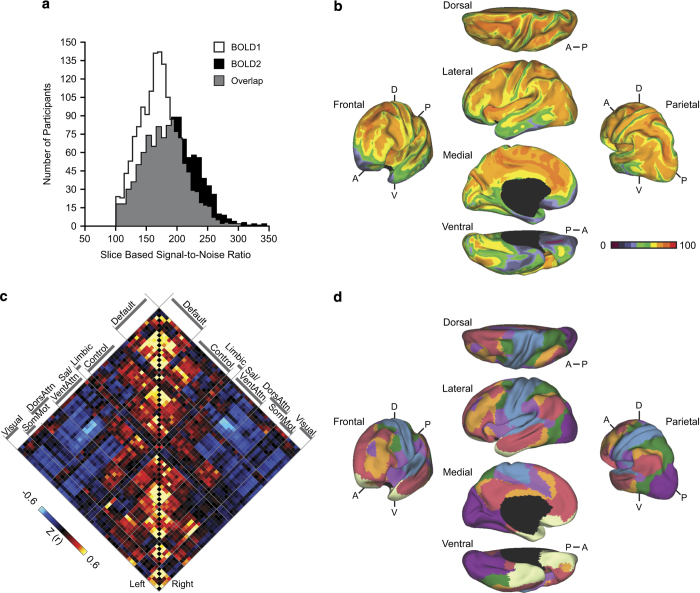
Functional measures of brain networks. (**a**) Histograms of mean slice-based temporal signal-to-noise (sSNR) values for the first and second rest runs illustrate variance in data quality across subjects. (**b**) The mean voxel-based temporal SNR map of the first rest run from the full sample (*n*=1,570) illustrates spatial variance in data quality across the cortical surface. The map is displayed for multiple views of the left hemisphere in Caret PALS space. A, anterior; P, posterior; D, dorsal; V, ventral. Note the regions of reduced SNR near to the sinuses and inner ear space. (**c**) A correlation matrix shows the complete coupling architecture of the full cerebral cortex measured at rest. Regions determined based on the 17-network solution from Yeo *et al.*^10^. Values reflect z-transformed Pearson correlations between every region and every other region. Within-network correlations fall along the diagonal displayed in the center. Between-network correlations are plotted away from the diagonal and reveal both positive (red) and negative (blue) correlations. (**d**) The functional network organization of the human cerebral cortex revealed through intrinsic functional connectivity. Colors reflect regions estimated to be within the same network. The approach groups similar correlation profiles based on a winner-take-all solution, with every surface vertex assigned to its best-fitting network^10^. The present data fully cover the striatum, thalamus, and cerebellum allowing for analyses that extend beyond the cerebral cortex (see Buckner *et al.*^11^ and Choi *et al.*^12^).

Data quality for the resting state scans was quantified through the Automated Functional MRI Quality Assessment Tool^35^. To facilitate quality assessment and data analyses a broad range of commonly used quantitative data quality metrics are included in the release dataset. Histograms of mean temporal sSNR values for the first and second rest runs are displayed in Fig. 2a. Histograms of number of relative movements in 3D space (>0.1 mm), and maximum absolute movement in 3D space (mm) for the first and second rest runs are presented in Supplementary Fig. 3a,b. Slice-based SNR was also used as exclusionary criteria. If the sSNR for the whole brain (mean sSNR over all slices within the brain mask weighted by the slice size) was less than 100 for the first BOLD run, all data from that participant were excluded from the release. If the temporal sSNR for the second BOLD run was less than 100, only that run was excluded. This means a participant could be included with a single BOLD run, when two runs were acquired, but the second run was lost due to data quality concerns.

Signal loss and susceptibility artifacts occur as a result of magnetic field inhomogeneities, potentially biasing or obscuring results from functional connectivity analyses. In T2*-dependent (BOLD) images, the decay in recoverable signal is exacerbated in regions where the brain is adjacent to air (e.g., sinus cavities)^51^. To estimate the topographic pattern of susceptibility artifacts in the present data we computed the voxel-level temporal SNR of the motion-corrected fMRI time series in each participant’s native volumetric space (the mean of the signal at each voxel over the BOLD run divided by the variance). The resulting voxel-level SNR was then projected to FreeSurfer surface space, averaged across the 1,570 subjects, and displayed in Caret PALS space (Fig. 2b)^52^. Clear spatial variation in voxel-level SNR was evident across the cortical mantle. As expected, decreased voxel-level SNR was pronounced in anterior aspects of inferior and medial temporal lobe, as well as in the orbital frontal cortex.

To provide an additional data quality metric, fractional Amplitude of Low Frequency Fluctuations (fALFF)^53,54^ were computed for each participant. fALFF reflects the total power in the low frequency range (0.01–0.08 Hz) of an fMRI image, normalized by the total power across all frequencies. fALFF has been theorized to suppress non-specific signal components in the resting-state fMRI, providing improved sensitivity and specificity to detect regional spontaneous brain activity. Histograms of mean fALFF for the first and second rest runs are displayed in Supplementary Fig. 4a. Voxel-level fALFF estimates were averaged across the 1,570 subjects, and displayed in Caret PALS space (Supplementary Fig. 4b)^52^.

The present data sample is of generally high quality because of the exclusion criteria. However, scan quality is not uniformly distributed across the sample. Factors such as head motion can systematically influence resting-state network measures^15,32–34^. To facilitate informed analyses of the available data, the sSNR, number of micro-movements, and maximum movements across several key group divisions are depicted in Supplementary Fig. 5. Particular care should be taken when selecting sub-populations that could bias results (for example splitting groups by sex or number of available BOLD runs).

### IQ, personality, and behavioral measures

Selected analyses of the available behavioral phenotypes are reported to highlight data quality, scale/measure validity, and potential analysis applications. The first analyses establish the validity of our online estimates and the sample characteristics for IQ. The analyses that follow explore personality assessments and then cognitive task performance.

**Figure 3:**
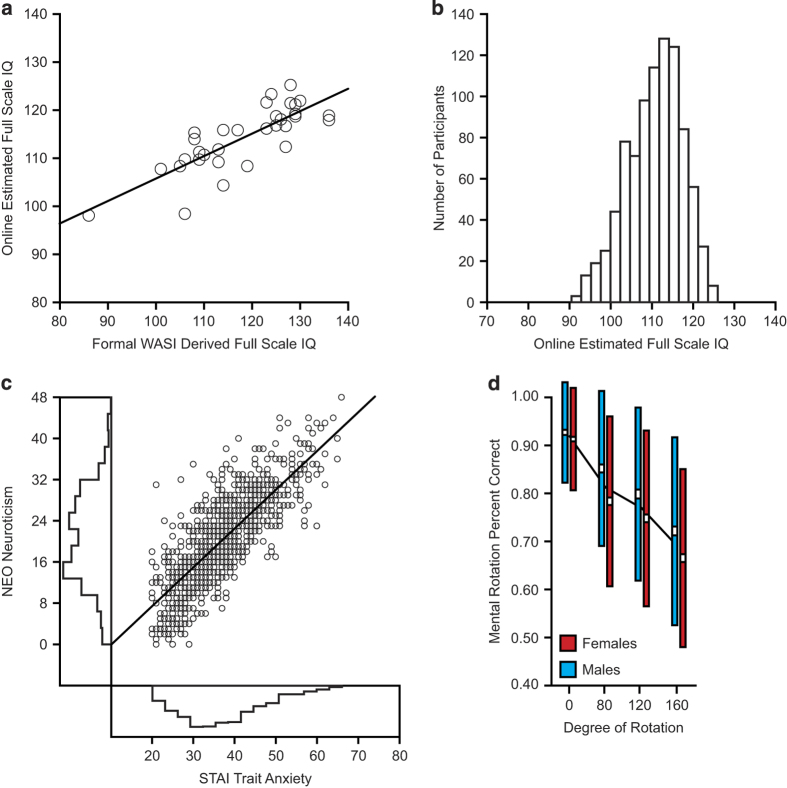
IQ, behavioral, and personality measures. (**a**) Online estimates of full scale IQ are consistent with standard Wechsler Abbreviated Scale of Intelligence (WASI) full-scale IQ estimates. Scatter plot reflects relation between average online and WASI estimates of full scale IQ (*n*=33; r=0.80). (**b**) Histogram reflects the distribution of the mean derived estimates of full scale IQ. Consistent with the sample recruitment from Boston area universities and colleges, MGH, and the surrounding communities, the mean estimated full scale IQ for the sample is 110.7±6.7. (**c**) Participants exhibit expected personality and temperamental characteristics. Scatter plot of available data reflects expected relations between STAI trait anxiety and NEO neuroticism. Histograms of anxiety and neuroticism are represented on the x and y axes respectively. (**d**) Graphs reflect mental rotation task performance for females and males. White boxes indicate standard error, colored boxes reflect standard deviation, and the black lines denote the sample mean for each condition. Performance decreases with more difficult rotations.

To estimate validity of the online IQ estimates, online estimates of full scale IQ were examined in relation to Wechsler Abbreviated Scale of Intelligence (WASI) derived estimates of full-scale IQ collected in person^55^. Thirty-three participants completed the WASI on the day of scan in addition to the full GSP online battery. A strong relation was found between the average estimated IQ from the WASI with that derived from the online estimates (r=0.80; Fig. 3a). As expected, the derived estimates of full scale IQ were normally distributed across the sample (Fig. 3b). Consistent with the sample recruitment from Boston area universities and colleges, MGH, and the surrounding communities, the mean estimated full scale IQ for the sample was elevated (110.7±6.7) relative to the expected distribution for the general population. Histograms reflecting the respective distributions of matrix reasoning and derived estimates of full scale IQ are presented in Supplementary Fig. 6.

Regarding personality estimates, participants exhibited the anticipated relations linking conceptually overlapping personality and temperamental characteristics. Consistent with a substantial literature on negative affect^56–58^, a strong association linked trait anxiety and neuroticism (r=0.80, *P*<0.001; Fig. 3c). Substantial co-variation exists across exploratory and disinhibitory behaviors, such as novelty seeking and impulsivity^59,60^. Analyses of the present data highlight the predicted relation between self-reported novelty seeking and impulsivity (r=0.62, *P*<0.001; Supplementary Fig. 7).

Cognitive task performance also suggests measurement validity. In the mental rotation task included in the initial release, participants were asked to compare two 3D objects and indicate if they were identical or mirror images of each other^61,62^. Since Shepard and Metzler^61^ first elaborated the concept, mental rotation has been a commonly used measure of spatial ability. In the mental rotation task, participants were presented with pairs of 3D, asymmetrical groupings of cubes. The relative rotation of each object pair in 3D space varied over the course of the experiment (0°, 80°, 120°, or 180°). Participants completed 9 trials for each rotation condition, 36 in total. In half of the available trials the shapes were identical or mirror images of each other. Participants’ performance was estimated based on their speed and accuracy to distinguish between the mirrored and non-mirrored pairs. As the extent of object rotation increased, participants displayed the expected decrease in performance (Fig. 3d)^61,62^. As predicted by prior evidence of sex differences in spatial processing^63,64^, the males in our sample exhibited increased mental rotation accuracy, relative to the females, across each non-0° rotation condition (ts>4.10; ps<0.001).

Cognitive control over information processing can be dynamically adjusted in response to environmental demands^65,66^. To establish an index of behavioral responses to shifting task demands participants completed a modified version of the Eriksen flanker task^65^. The flanker task requires the participant to focus on a given stimulus while inhibiting attention to flanking stimuli, providing estimates of both attentional and inhibitory control. In the included flanker task, participants were presented with groups of 5 arrows pointing left or right. They were instructed to respond to the center arrow. When the arrows were printed in green font participants responded in the same direction as the middle arrow. When the arrows were printed in red font participants responded in the opposite direction of the middle arrow. Participants completed 192 flanker trials, with 12 trials in each block. Over the course of the task participants completed 8 switch and 8 non-switch blocks. In switch blocks the color of the presentation alternated between red and green font throughout the block. As expected, the increased demand on selective visual attention and inhibition in the switch blocks resulted in decreased accuracy and increased response times, relative to non-switch blocks (ts>25.51, ps<0.001; Supplementary Fig. 8).

### Analysis applications

Selected analyses of the anatomical data are reported to illustrate (1) the potential of the available data through a typical use case that partials out nuisance variables and (2) a brain-behavior relation that requires a large sample size to detect.

A well-defined amygdala-medial prefrontal cortex (mPFC) circuit contributes to emotional processes^67–70^. Subtle shifts within the anatomy of this circuit, present in the general population, have been reported to track with the expression of negative affect in a subset of the present data^13^. To examine the presence of these relations in the formal GSP release sample, analyses were conducted mirroring those in the recent Holmes *et al.* ^13^ publication (*n*=897). Due to partially overlapping data, these analyses should not be interpreted as a true replication of the observed effect. Briefly, trait negative affect was computed as the average of the Z-scores for five self-report measures associated with the experience of negative affect^56–58^. These scales included the trait form of the Spielberger State/Trait Anxiety Inventory^71^, the neuroticism scale from the NEO five-factor inventory^72^, the behavioral inhibition component of the Behavioral Inhibition/Behavioral Activation Scale^73^, the total mood disturbance score from the Profile of Mood States^30^, and the harm avoidance scale from the Temperament and Character Inventory^74^. Block linear regressions were conducted separately for both the left and right amygdala. Analyses partialed out the variance associated with site, console software version, estimated IQ^75^, age, sex, and ICV and then examined the relation between amygdala volume and negative affect. Given prior evidence suggesting opposing relations in the amygdala and the mPFC with negative affect, surface-based cortical thickness analyses were conducted on the FreeSurfer parcelation of the region labeled by Desikan *et al.* ^76^ as the rostral anterior cingulate. Block linear regression partialed out the variance associated with site, console software version, estimated IQ^75^, age, and sex and then examined the relation between mPFC cortical thickness and negative affect.

Analyses revealed slight, yet opposing structural differences in the amygdala and medial prefrontal cortex in the present sample of young adults. Consistent with its hypothesized role in anxiety and affective illnesses, amygdala volumes co-varied with negative affect (left: F_1,889_=11.36; *P*<0.001; r=0.11; Supplementary Fig. 9a; right: F_1,889_=4.34; *P*<0.05; r=0.07). In line with the suggested role of the mPFC in the downregulation of amygdala activity, reduced left hemisphere rostral anterior cingulate cortical thickness associated with subtle increases in negative affect (F_1,890_=4.73; *P*<0.05; r=−0.07; Supplementary Fig. 9b).

Impairments in affective experience are hypothesized to result from a breakdown in the interactions between subcortical and cortical structures^77,78^. To further examine how the correlation between amygdala volume and mPFC thickness associates with negative affect, the sample was split into groups with low-medium (*n*=760), and high (*n*=137) negative affect. High and low-medium groups were defined as one standard deviation above or below the mean negative affect score (0.00±0.83). No detectable relation was observed between amygdala volume and mPFC thickness in the low-medium negative affect participants (F_1,753_=0.886; *P*=0.347; r=0.03; Supplementary Fig. 9c). A negative correlation between left amygdala volume and mPFC thickness was evident among individuals reporting the most extreme negative affect (F_1,130_=3.84; *P*=0.05; r=−0.17; Supplementary Fig. 9d). The amygdala-mPFC correlation in the high negative affect participants was significantly different from the relation observed in the remaining participants (Z=2.19, *P*<0.05).

To mitigate spurious effects resulting from population admixture and cultural biases in self-reported affect^79^, the original Holmes *et al.* ^13^ analyses were restricted to white non-Hispanic participants of European ancestry. When considering these participants (*n*=566) in the current sample, negative affect co-varied with amygdala volumes (left: F_1,558_=9.57; *P*<0.005; r=0.13; right: F_1,558_=4.04; *P*<0.05; r=0.09) and was associated with decreases in mPFC thickness (F_1,559_=3.94; *P*<0.05; r=−0.08). When dividing the participants into low-medium (*n*=473) and high (*n*=93) negative affect, no detectable relation was observed between amygdala volume and mPFC thickness in the low-medium negative affect participants (F_1,466_=0.086; *P*=0.769; r=0.01). An inverse correlation between left amygdala volume and mPFC thickness was evident among the individuals with the most extreme negative affect (F_1,86_=3.96; *P*<0.05; r=−0.21).

### Analysis of functional network properties

Estimates of intrinsic functional coupling can be used to explore brain organization^80^ as well as the basis for graph theoretical analyses of network properties^81^. To illustrate the current data’s utility for such analyses, we estimated a cortical functional coupling matrix across all available region pairs based on the functional atlas of Yeo *et al.* ^10^; (see also Power *et al.* ^82^). This matrix is a comprehensive description of the correlation strength of all region pairs across the cortex for the complete dataset of 1,570 participants (Fig. 2c). This matrix or similar matrices derived from subsets of participants can provide a powerful means to explore relations between network properties and function.

One caveat in interpreting the magnitude of functional correlations is that the correlation structure of resting-state data is inherently biased by a nonuniform distribution of SNR^51^. This point should be carefully considered when using the present data. To illustrate this caveat, we assessed the reliability of the correlation estimates using the test-retest data. Consistent with the observed spatial variation in SNR across the cortical mantle (Fig. 2b), estimates of intrinsic functional coupling are not uniformly reliable across the cortex (Supplementary Fig. 10). Decreased test-retest reliability was particularly evident in the ‘Limbic network,’ encompassing aspects of orbital frontal and inferior medial prefrontal cortex as well as portions of temporal pole. This analysis is a reminder that spatial variation in signal quality across the brain should be considered in all analyses of functional coupling derived from BOLD data.

As a final illustration of how the functional coupling can be used to derive network properties, the topographic organization of the human cerebral cortex across both rough and fine-grained resolutions was estimated from the coupling of each vertex across the entire cortical mantle mimicking Yeo *et al.* ^10^ (Fig. 2d; Supplementary Fig. 11; Supplementary Fig. 12). Other approaches can be productively applied to these data^83,84^.

## Usage Notes

Large-scale imaging datasets are necessary to address complex questions regarding the relation between brain and behavior. The GSP release data provides a carefully vetted collection of neuroimaging, behavioral, cognitive, and personality data for 1,570 participants. The data collection and anonymization procedures employed in the GSP have resulted in a dataset that is highly suitable for processing, with minimal restrictions and without imposed dependencies on proprietary tools. The conversion of the available neuroimaging data from raw to NIfTI-1 file format, data anonymization, and the quantitative estimate of data quality were implemented through publicly accessible processing tools.

## Additional Information

**How to cite this article:** Holmes, A. J. *et al.* Brain Genomics Superstruct Project initial data release with structural, functional, and behavioral measures. *Sci. Data* 2:150031 doi: 10.1038/sdata.2015.31 (2015).

## Supplementary Material



Supplementary Figures

Supplementary Appendix A

Supplementary Appendix B

Supplementary Appendix C

Supplementary Appendix D

## Figures and Tables

**Table 1:** MRI Acquisition Details.

	**T1 MEMPRAGE**	T2* BOLD
Duration	2’12’’	6’12’’
TR (sec)	2.2	3.0
TE (msec)	1.5/3.4/5.2/7.0	30
Flip angle (°)	7	85
TI (sec)	1.1	n/a
Orientation	Sagittal	T>C-12.5
Slice Thickness (mm)	1.2	3.0
Slice number	144	47
Resolution (pixels, mm)	1.2×1.2×1.2	3.0×3.0×3.0

**Table 2:** Demographic characteristics and available phenotypes for the data release sample

	**BOLD**		**Behavioral**	
	**One Run**	**Two Runs**	**Self-Report Only**	**All Behavioral**
n	1570	1139	926	892
Age	21.5±2.9	21.2±2.7	21.6±2.9	21.6±2.8
Age Range	18–35	18–35	18–35	18–35
% Female	57.6	59.0	57.9	57.4
Education (Yrs.)	14.5±1.9	14.3±1.8	14.59±2.0	14.6±1.9
% Right Handed	92.3	90.3	91.6	91.7
% White (not Hispanic or Latino)	61.6	62.7	63.6	63.7
Notes: Education is presented as the mean for the group. However, many participants were still in school at the time of enrollment so current education level should not be taken as a proxy for socioeconomic status or ultimate educational attainment.				

**Table 3:** Participants by Age and Sex.

**Age (2-year bin)**	**Males n**	**Females n**
18–19	229	331
20–21	220	289
22–23	114	149
24–25	41	68
26–27	26	40
28–29	14	14
30–31	13	7
32–33	6	5
34–35	2	2

**Table 4:** Structural Phenotype Reliability

**MRI Variable**	**Pearson Correlation (r)**
*Global*	
Intracranial Volume	0.999
Brain Volume (including ventricles)	0.996
Brain Volume (without ventricles)	0.996
Right Cortical Thickness	0.894
Left Cortical Thickness	0.899
Right Cortical Surface Area	0.998
Left Cortical Surface Area	0.999
	
*Regional Volumes*	
Posterior Corpus Callosum	0.992
Middle Posterior Corpus Callosum	0.956
Central Corpus Callosum	0.952
Middle Anterior Corpus Callosum	0.948
Anterior Corpus Callosum	0.980
Right Amygdala Volume	0.837
Left Amygdala Volume	0.931
Right Hippocampal Volume	0.968
Left Hippocampal Volume	0.959
	
*Regional Thicknesses*	
Right Medial Prefrontal	0.746
Left Medial Prefrontal	0.779
Right Caudal Middle Frontal	0.820
Left Caudal Middle Frontal	0.837
Right Lateral Occipital	0.902
Left Lateral Occipital	0.872
Right Lingual	0.842
Left Lingual	0.845
Right Caudal Anterior Cingulate	0.765
Left Caudal Anterior Cingulate	0.849
Right Posterior Cingulate	0.803
Left Posterior Cingulate	0.878
Note: All correlations are significant at the 0.01 level (2-tailed); MRI variable definitions detailed in Supplementary Appendix D. Right and Left refer to the right and left hemisphere.	
