# Supplementary material for: Brain Genomics Superstruct Project initial data release with structural, functional, and behavioral measures
Source: Sci Data. 2015 Jul 7;2:150031. doi: 10.1038/sdata.2015.31 (PMC4493828; doi:10.1038/sdata.2015.31)
Supplement: Supplementary Figures [file sdata201531-s2.pdf]

Brain Genomics Superstruct Project initial data release with structural, functional, and behavioral measures: supplementary figures

Avram J. Holmes[abcd], Marisa O. Hollinshead[abd], Timothy M. O'Keefe[a], Victor I. Petrov[a], Gabriele R. Fariello[ac], Lawrence L. Wald[d], Bruce Fischl[d], Bruce R. Rosen[d], Ross W. Mair[a,d], Joshua L. Roffman[cd], Jordan W. Smoller[c], and Randy L. Buckner[abcd]

a. Center for Brain Science, Harvard University, Cambridge, MA 02138
b. Department of Psychology, Harvard University, Cambridge, MA 02138
c. Department of Psychiatry, Massachusetts General Hospital and Harvard Medical School, Boston, MA 02114
d. Athinoula A. Martinos Center for Biomedical Research, Department of Radiology, Massachusetts General Hospital and Harvard Medical School, Charlestown, MA 02129

Table of Contents

Supplementary Figure 1 ____ 2
Supplementary Figure 2 ____ 3
Supplementary Figure 3 ____ 4
Supplementary Figure 4 ____ 5
Supplementary Figure 5 ____ 6
Supplementary Figure 6 ____ 7
Supplementary Figure 7 ____ 8
Supplementary Figure 8 ____ 9
Supplementary Figure 9 ____ 10
Supplementary Figure 10 ____ 11
Supplementary Figure 11 ____ 12
Supplementary Figure 12 ____ 13

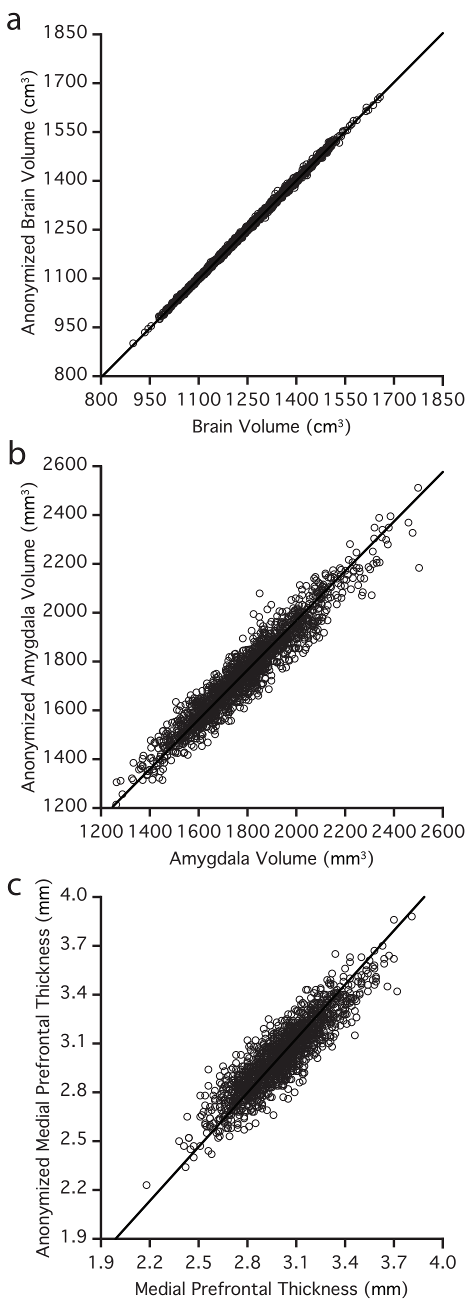

Supplemental Figure 1. Face blurring of the structural MRI data induces noise into morphometric estimates. Scatter plots display the correlations between morphometric estimates derived from face blurred and raw anatomical scans across estimated (a) brain volume, (b) amygdala volume, and (c) medial prefrontal cortical thickness. Estimated values were defined using FreeSurfer version 4.5.0 (see Methods). The medial prefrontal subregion (c) includes portions of the left rostral anterior cingulate cortex and the subgenual anterior cingulate cortex.

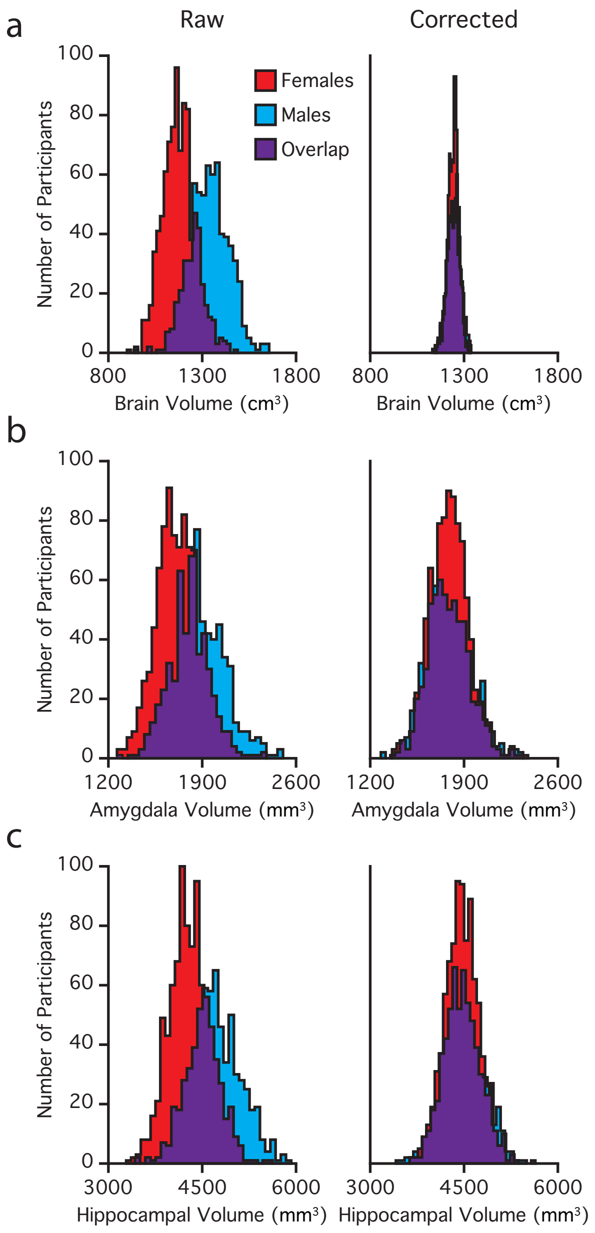

Supplemental Figure 2. Atlas normalization corrects for head size variation in regional and whole-brain morphometric analyses. Histograms of brain (a), amygdala (b), and hippocampal (c) volumes are reported for females and males. Graphs on the left reflect raw morphometric estimates. Graphs on the right reflect values after correcting for estimated intracranial volume[40].

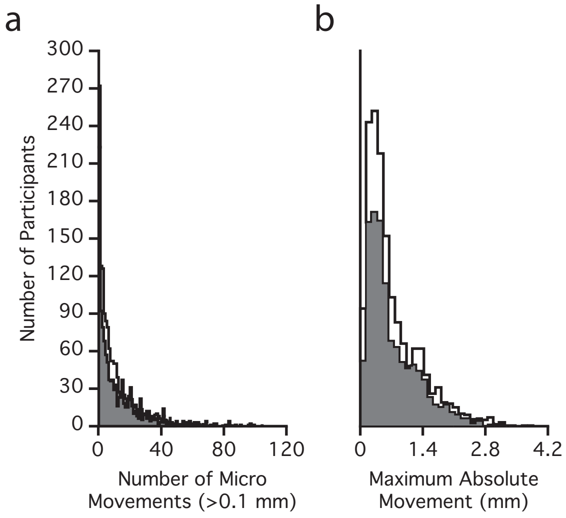

Supplemental Figure 3. Additional measures of functional rest scan data quality. Histograms of (a) number of relative movements in 3D space (>0.1mm), and (b) maximum absolute movement in 3D space (mm) for the first and second rest runs.

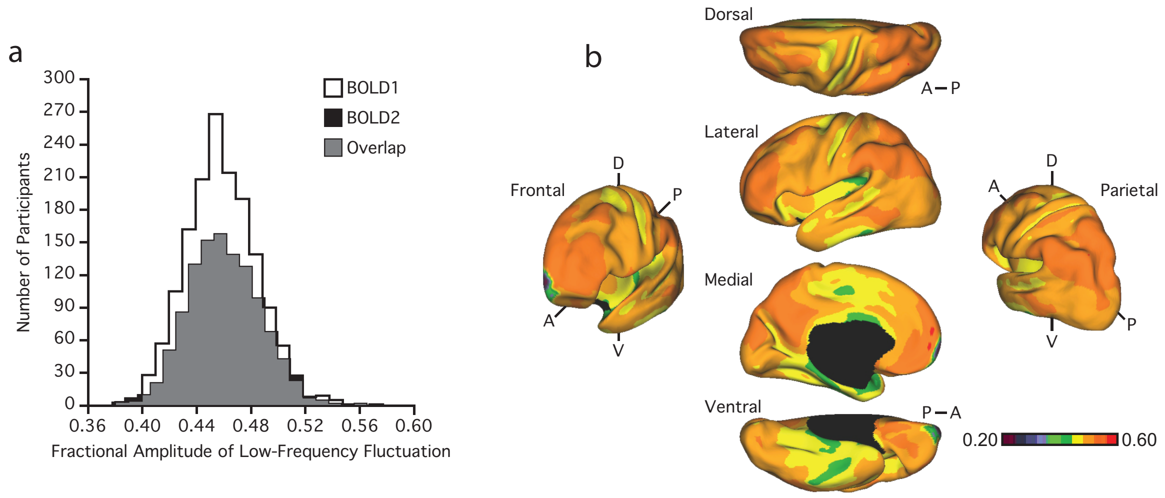

Supplemental Figure 4. Estimated fractional Amplitude of Low Frequency Fluctuations. (a) Histograms of mean fractional Amplitude of Low Frequency Fluctuations (fALFF) values across subjects. (b) The mean voxel-based fALFF map of the first rest run from the full sample (n=1,570) illustrates spatial variance in data quality across the cortical surface. The map is displayed for multiple views of the left hemisphere in Caret PALS space. A, anterior; P, posterior; D, dorsal; V, ventral. Note the regions of reduced fALFF near temporal and orbital frontal cortex echo reduced SNR near to the sinuses and inner ear space (Fig. 2b).

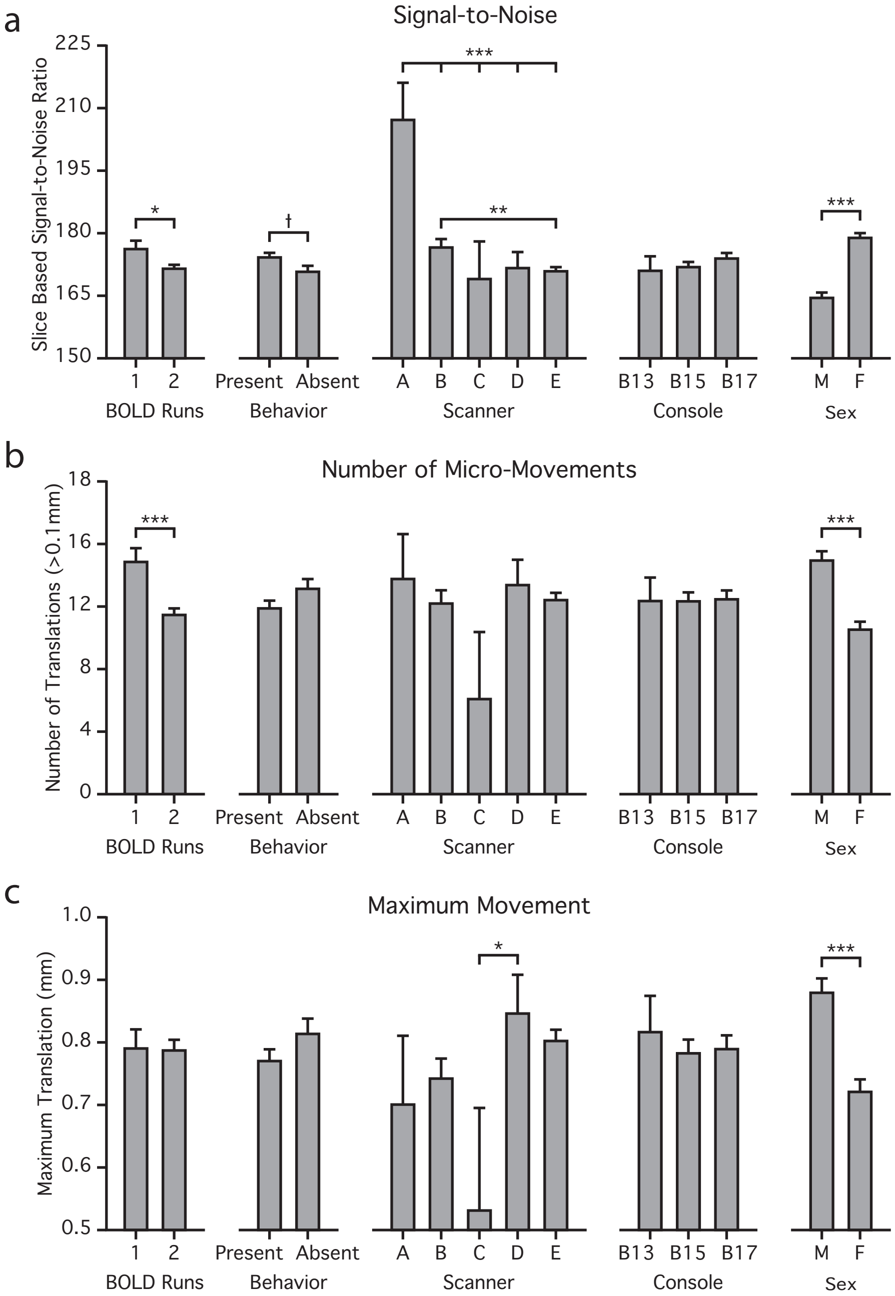

Supplemental Figure 5. Scan quality is not uniformly distributed across the sample. Figures reflect the mean and standard deviation for (a) mean slice based signal-to-noise values, (b) number of relative movements in 3D space (>0.1mm), and (c) maximum absolute movement in 3D space (mm) for the first rest run from the full sample (n=1,570). From left to right each graph reflects data quality for participants with one or two rest runs, individuals with and without available behavioral data, the scanner/site of acquisition, the console version at the date of scan, and for each sex.

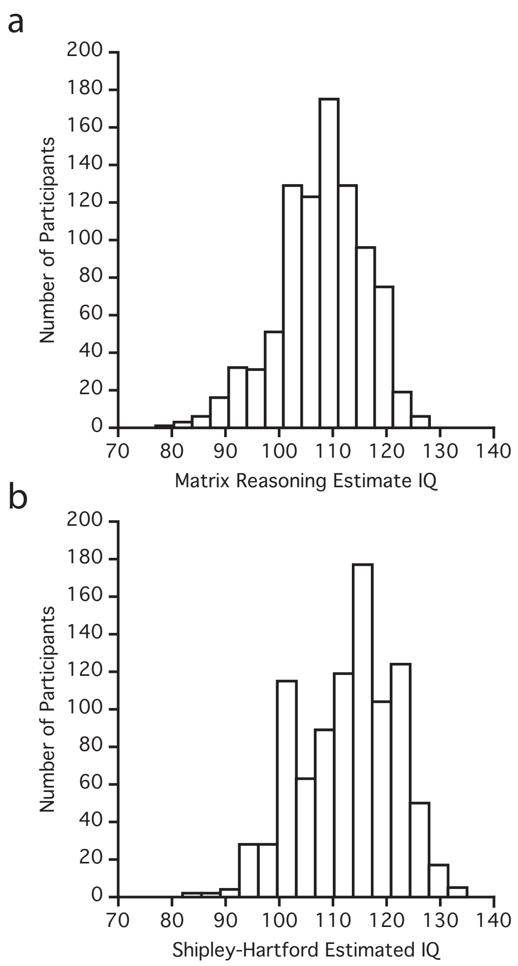

Supplemental Figure 6. Distribution of online estimated full scale IQ. Histograms reflect (a) matrix reasoning, (b) and Shipley derived estimates of full scale IQ.

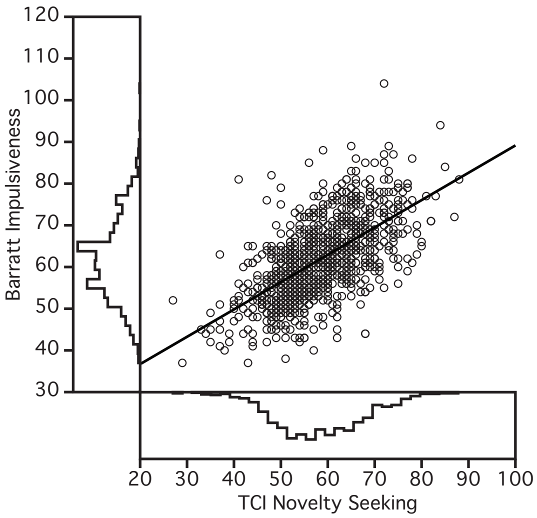

Supplemental Figure 7. Participants exhibit expected personality characteristics. Scatter plot of available data reflects expected relations between TCI novelty seeking and Barratt Impulsiveness. Histograms of both novelty seeking and impulsiveness are represented on the x and y axes respectively.

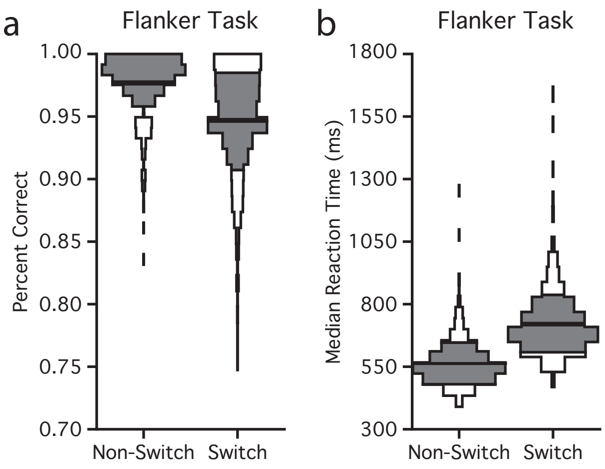

Supplemental Figure 8 Participants exhibit expected behavioral characteristics. Graphs reflect Flanker task (a) percent correct and (b) reaction time across blocks of non-switch and switch trials. Data are presented as histograms. Black and grey areas reflect standard error and standard deviation respectively.

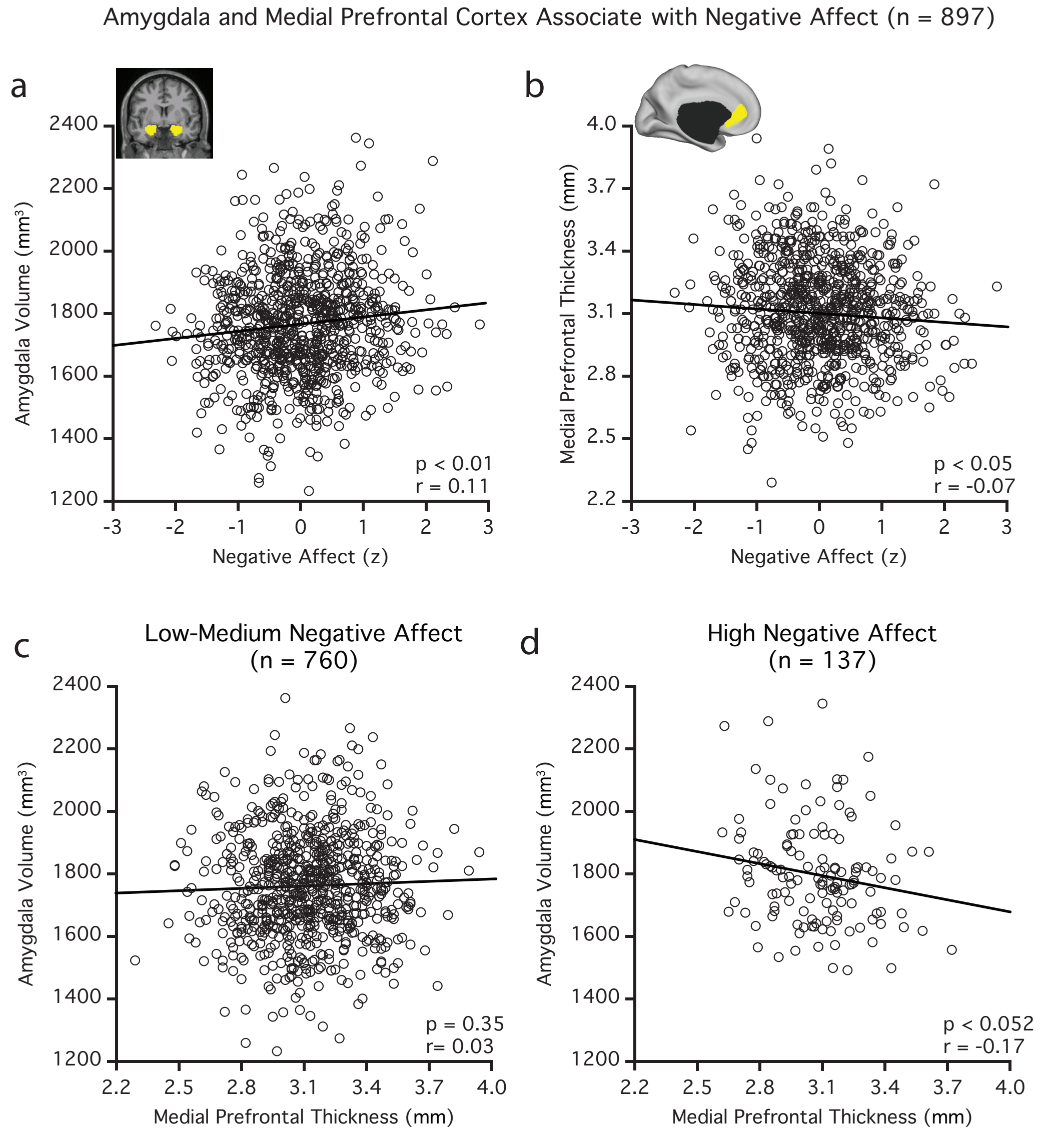

Supplemental Figure 9. Opposing structural differences in the amygdala and medial prefrontal cortex are present in the young adults with the most extreme negative affect. The scatter plots display the correlation between trait negative affect and left (a) amygdala volume and (b) medial prefrontal cortical thickness (mPFC), as well as representative segmentations of the left amygdala and mPFC. Scatter plots (c,d) represent the distribution of values for the left amygdala volumes and left medial prefrontal thickness estimates for the (c) low-medium and (d) high negative affect groups. Reported r values reflect Pearson correlations after partialing out variance associated with collection site, scanner software, estimated IQ, age, and sex. Estimated intracranial volume was additionally partialed from the amygdala volume estimate.

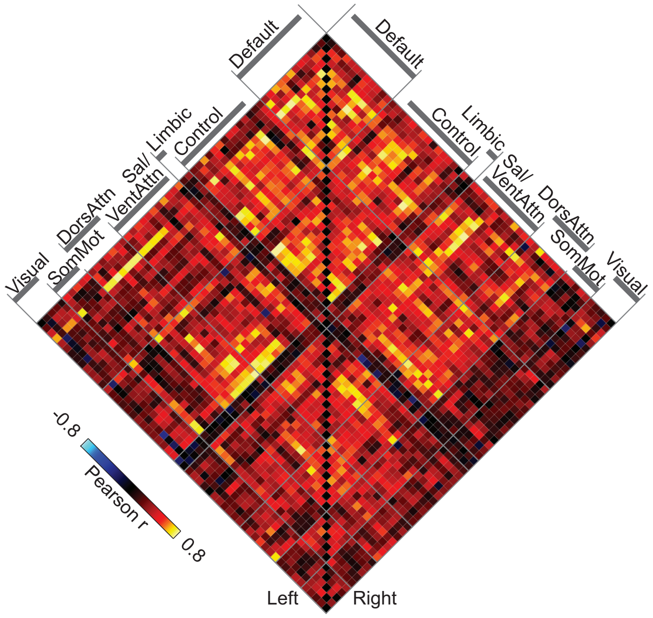

Supplemental Figure 10. Estimates of intrinsic functional connectivity are not uniformly reliable across the cortex. The correlation matrix shows the complete test-retest reliability of the full cerebral cortex measured at rest (n=69). Regions determined based on the 17-network solution from Yeo et al. (2011)[10]. Values reflect Pearson correlations of the correlation values between visits 1 and 2 where independent data were acquired.

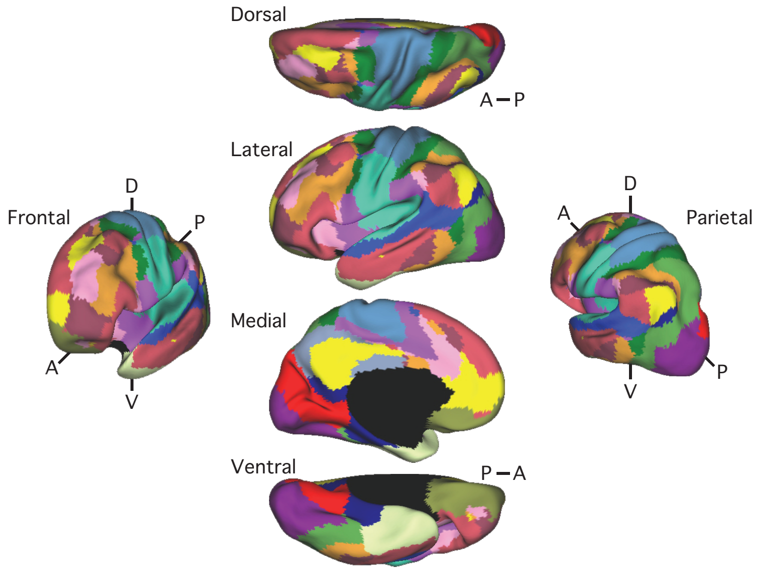

Supplemental Figure 11. The topographic organization of the human cerebral cortex estimated by intrinsic functional connectivity. A fine-resolution 17-network parcellation of the human cerebral cortex based on 1,570 participants. These results generalize those of Yeo et al.[10]. Colors represent regions that are estimated to be within the same network based on similarity in correlation profiles. The approach, which represents just one of many different ways to analyze functional correlations, uses a winner-take-all solution such that every vertex on the surface is assigned to its best-fitting network.

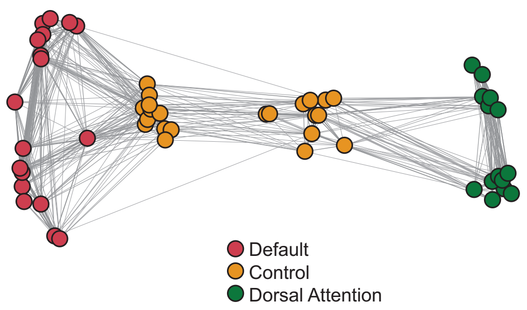

Supplemental Figure 12. Intrinsic functional coupling provides information about between-network interactions. Spring-loaded graphs show selected nodes of the frontoparietal control network, dorsal attention network, and default network. Regions determined based on the Yeo et al., 2011 17-network solution[10]. Note the clear separation of two networks that are known to be negatively correlated (anticorrelated) with one another (the Default Network and Dorsal Attention Network). Nodes of the Frontoparietal Control Network show coupling with both networks, with subsets of nodes aligning more to one network or the other, consistent with the notion that the Frontoparietal Control Network interacts with adjacent networks. A challenge in such analyses is spatial blurring of the low-resolution functional MRI data, which may induce differences in coupling strengths between adjacent regions. Spatial blurring should be considered in analysis of network properties of the present and similar datasets.
