# Supplementary material for: Brain Genomics Superstruct Project initial data release with structural, functional, and behavioral measures
Source: Sci Data. 2015 Jul 7;2:150031. doi: 10.1038/sdata.2015.31 (PMC4493828; doi:10.1038/sdata.2015.31)
Supplement: Supplementary Appendix A [file sdata201531-s3.pdf]

CONSENT TO PARTICIPATE IN NEUROIMAGING RESEARCH:
Brain Genetics Study
Healthy Volunteers for Add-on Scans

Please consider this information carefully before deciding whether to participate in this research.

Purpose of the research:
The purpose of this study is to promote our understanding of the effects of genes on brain structure and function. All living things are made of cells. Genes are the part of cells that contain the instructions that tell our bodies how to grow and work, and determine characteristics such as hair and eye color. Genes are inherited (that is, passed from parent to child). DNA is the material (chemical letters) that makes up your genes. Three thousand healthy volunteers will participate in this study. This study is being added on to the study you are already participating in. You do not have to participate in this additional study. If you do choose to participate, this additional study will add approximately 20 to 40 minutes to your time in the MRI scanner and you will complete an additional 20 minutes of forms before or after the MRI session. You may be asked to participate in cognitive tasks. Some of these tasks may be done on the day of your appointment while others can be done online. The optional online cognitive tasks will take up to 3 hours and can be completed from home at any time. If you choose to provide a phone number on the re-contact form, a follow-up call will be made to discuss your experience with the online portion of our study.

What you will do in this research:
<u>MRI</u>
Pictures of the shape and function of your brain will be taken while keeping your eyes open. The scanning itself will last approximately 20 to 40 minutes. Additionally, you will be asked to fill out some demographic forms and questionnaires before and/or after the scan. Some of these questionnaires will ask you about your personality and day-to-day behavior. We may also ask you fill out questionnaires about your social interactions with friends and family members. Furthermore, women will be asked about their hormonal patterns including questions regarding their estrogen levels. Because of the effects of medication and mental illness on the brain, you will be asked a series of questions about whether you are presently taking or have ever taken medication for depression, anxiety, and other forms of mental condition (e.g., schizophrenia). You will be asked about any history of neurological and psychiatric illness. If you have previously taken such medications or are currently taking any you should not participate in this study. If you feel uncomfortable in discussing such information, you should not participate. You do not need to tell us why you have chosen not to participate.

<u>Genetic Analysis</u>
In order to collect DNA that provides genetic information, you will be asked to spit into 1-2 saliva collection cup(s). After your DNA has been analyzed, we will freeze any “leftover” DNA and send it to the Center for Human Genetics Research (CHGR) located at Massachusetts General Hospital. We will assign your sample with a code number and staff at the CHGR will store it in a freezer. They will not be given your name or other information that could identify you with your sample. There is no scheduled date on which your samples and information in the bank will be destroyed. Your samples may be stored for research until they are “used up.”

With the exception of your DNA samples, all of the data collected as part of this study (genotypes, brain images, cognitive, and diagnostic/demographic information) will be shared with the investigator of the study that initially brought you to the Northwest Science Building today. This will include personally identifying information so that the investigator that referred

you to this study is able to link the data we provide with the data they collect. **No one other than members of the current study staff will be able to connect the DNA samples to your personally identifying information.**

Your **coded** genotypes, brain images, cognitive, and demographic information will be shared with other research investigators within and outside of Harvard (e.g., MGH). The coded information will not contain your name or other information that could likely identify you, and investigators will not be given any information that could link the coded information with identifying information.

A coded portion of your DNA sample will be sent to the Broad Institute for whole genome analysis. Usually researchers study a few areas of your genetic code that are linked to a disease or condition. In our whole genome analysis, we will be using all or most of your genes for research related to the brain and individual differences in the brain ranging from typical variation to psychiatric illnesses. The information will be sent with only a code number attached, and your name or other identifiable information will never be given.

In order to allow researchers to share test results, the National Institutes of Health (NIH) and other central repositories have developed special data (information) banks that collect the results of whole genome studies. The NIH or other data store genetic information and give it to other researchers to do more studies. We do not think there will be further risks to your privacy and confidentiality by sharing your whole genome analysis with these databanks; however, we cannot predict how genetic information will be used in the future. The information is sent with only a code number attached, and your name or other identifiable information is never given to them. There are safeguards in place to protect your information while it is stored in repositories and used for research.

Research using your whole genome information is important for virtually all disease and conditions. Therefore, the NIH data bank will provide study data for researchers working on any disease, which could include conditions such as Alzheimer's disease, mental illness, cancer, and others.

Cognitive Testing

You will be asked to complete a series of cognitive tasks (thinking processes) on any computer with internet access. In order to access the tests, you will be provided with a unique ID code that you will enter into a specific website. The online testing is expected to take up to 3 hours to complete.

The results from this study will be pooled with those of other similar studies and shared among researchers or used for teaching purposes. For example, a researcher at another institution may wish to reexamine the results of this study. When results are shared, only a code number identifies them. At no time will any identifying information such as your name be shared. Please choose not to participate in this study if you are uncomfortable with your coded data being shared.

In some cases, we may be interested in re-contacting you for additional information or to participate in a follow-up experiment. If we do, your participation is completely optional and you would be compensated appropriately for your time. If you would prefer that we refrain from re-contacting you, please initial below to indicate this.

Initial if you would prefer NOT to be re-contacted following this study: _________

Time required:

This study takes up to 1.5 hours (approximately 20 to 40 minutes of scanning plus paperwork) at the Northwest Science Building scanning facility, located at Harvard University. Approximately 3 hours are requested for the optional take-home online computer tasks.

Risks:

<u>MRI</u>

There are no known or foreseeable risks or side effects associated with scanning procedures beyond those that have already been discussed as part of your primary study.

<u>Genetic Analysis</u>

Saliva collection (i.e. spitting) is generally considered safe and painless. The main risk of allowing us to store and use your samples and certain limited health information for research is a potential loss of privacy. However, we will take the following steps to protect your privacy:

- We will store your samples only with a code.
- Information that could be used to identify you will only be shared with researchers within Harvard who have approval of the Committee on the Use of Human Subjects in Research (CUHS). This committee is a group that independently reviews and watches over all research studies involving people at Harvard University. The committee follows state and federal laws and codes of ethics to make sure that the rights and welfare of people taking part in research studies are protected.
- Information that likely could be used to identify you will never be shared with researchers outside Harvard.

Genetic information that results from this study does not have medical or treatment importance at this time. The study is conducted for research purposes only, therefore, we will not be sharing genetic information with you.

<u>Cognitive Testing</u>

There are no known or foreseeable risks or side effects associated with the cognitive tasks being administered in the study. Occasionally individuals may experience fatigue; therefore, we have allocated designated resting breaks throughout the online portion of this study.

Benefits:

At the end of the study, we will provide a thorough explanation of the study and of our hypotheses. We will describe the potential implications of the results of the study both if our hypotheses are supported and if they are disconfirmed. If you wish, you can send an email message to and we will send you a copy of any manuscripts based on the research (or summaries of our results).

Compensation:

You will receive payment of $50 for the scan and saliva sample. You will receive an additional $60 after completing the computer based take-home portion. You may also participate for course credit (1 credit per hour) in replacement of monetary payment. Monetary payments will be made by check through the mail and we will need your social security number to process the payment. In addition, please expect your check payment within 4-6 weeks of participation.

Confidentiality:

Your participation in this study will remain confidential, and your identity will not be stored with your data. Your responses will be assigned a code number, and the list connecting your name with this number will be kept in a locked room. When data are shared, your name will never be shared.

Participation and withdrawal:
Your participation in this study is completely voluntary, and you may refuse to participate or withdraw at any time without penalty or loss of benefits to which you are otherwise entitled.. Furthermore, you may skip any questions you do not want to answer. You will receive full payment for the MRI session and saliva sample even if you withdraw early. The online tasks are divided into 3 sections; you will be paid for partial completion of the online tasks in the amount of $20 or 1 course credit for each section.

You are allowed to withdraw your saliva sample at anytime within 1 month of your saliva collection date. However, once the DNA has been extracted and placed in the repository we will not be able to remove your sample. Please use the contact information listed below to notify us if you wish to withdraw your saliva sample from our study.

To Contact the Researcher:
If you have questions about this research, please contact

Whom to contact about your rights in this research, for questions, concerns, suggestions, or complaints that are not being addressed by the researcher, or research-related harm:

If you are injured during the course of the study and as a direct result of this study, you should contact the investigator at the number provided. Although compensation is not available, Harvard will assist you in obtaining medical treatment, including first aid, emergency treatment, and follow-up care as needed. Your insurance carrier should be billed for the cost of such treatment. If your insurance carrier denies coverage, Harvard is under no obligation to pay for the treatment but may do so in its sole discretion. By providing financial or other assistance, neither Harvard nor the researchers are stating that they are legally responsible for the injury.

Further information regarding compensation for injured research subjects may be obtained from the Committee on the Use of Human Subjects at the above number.

Agreement:
The nature and purpose of this research have been sufficiently explained and I agree to participate in this study. I understand that I am free to withdraw at any time without incurring any penalty.

Subject Signature: ____________________________________ Date: _________________

Subject Name (print): ____

Researcher Signature: ___________________________________ Date: ________________

Subject Identification

Protocol Title: Effects of Genotype on Brain Structure and Function

Principal Investigator:

Site Principal Investigator:

Description of Subject Population: Healthy Volunteers for Add-On Scans

Collection of Samples and Health Information for Research

About this consent form

This form contains important information. If you have any questions about the research or this form, please ask us. If you decide to take part in this research study, you must sign this form.

Why is this research study being done?

The purpose of this study is to understand the effects of genes on brain structure and function. Genes, which are inherited, are the part of cells that instruct our bodies how to grow and work, and determine characteristics such as eye color. DNA is the material that makes up your genes. Five thousand healthy volunteers will participate in this study.

What will happen in this research study and how long will it take?

This study is in addition to the study you are already participating in and will add 20 to 40 minutes in the MRI scanner. You will be asked to keep your eyes open while pictures of your brain are taken. You will also be asked to fill out forms that may ask you questions about your age, race, general health and educational background. You may skip any questions you do not feel comfortable answering. If you choose to provide a phone number on the re-contact form, a followup call will be made to discuss your experience with the online portion of our study.

<u>Genetic Analysis</u>
You will be asked to spit into 1-2 saliva collection cup(s). DNA will be removed from the collected sample and typed for genes that may play a role in brain function.

<u>Cognitive Testing</u>
You will be asked to complete 3 hours of online cognitive tasks from any computer with internet access. A computer will be available at the Martinos Center. If you do not have internet access and are unable to complete the online cognitive tasks at the Martinos Center, we will provide a

portion of the cognitive tasks as a take home paper version along with a self-addressed, pre-stamped envelope which should take 2 hours to complete.

What is a tissue bank and what is the purpose of storing my "leftover" DNA in a research tissue bank?

Our research tissue bank is located at the MGH Center for Genetics Research (CHGR) located in the Simches Research Building. The purpose of this research tissue bank is to collect, process, and store information about DNA until researchers need to study questions related to the brain and diseases of the brain; for example, what causes, helps prevent, treat, or cure a disease, and how it may be passed on in families. Tissue banks have rules about which researchers can get samples and what kind of research they can do using the samples.

We will freeze any "leftover" DNA and send it to the MGH CHGR. Your name or other identifying information will not be kept with your sample. Your sample will be assigned a code number to connect it to your personal and health information. This information will be stored in a password protected computer database. Only research staff will know the password.

We are asking your permission to freeze and store your samples in a tissue bank and to store some of your health information with your samples. We do not plan on re-contacting you **unless** the DNA collection fails or your DNA sample runs out.

May we re-contact you to request another DNA sample for the reasons just indicated?
☐ **Yes** ☐ **No** **Subject Initials ___________**

If a new study is developed we would like your permission to re-contact you. At any time you may request that we stop contacting you.

May we contact you in the future to ask your interest in participating in future studies?
☐ **Yes** ☐ **No** **Subject Initials ___________**

For what type of research will my samples and imaging data be used?

We are interested in how genes affect the brain in healthy people and those with neuropsychiatric illnesses. In healthy people we may look at how genes that are thought to play a role in brain development affect brain structure and function. We may also examine how genes that have been linked to mental and neurologic illness affect the brain, both in healthy individuals and in people who have been diagnosed with these disorders.

Which researchers can use my samples and imaging data and what information about me can they have?

With the **exception** of your DNA samples, all of the data collected as part of this study (genotypes, brain images, cognitive, and diagnostic/demographic information) will be shared with the investigator of the study which initially brought you to the Martinos Center today including personally identifying information. **No one other than members of the current study staff will be able to connect the DNA samples to your personally identifying information.**

Your **coded** genotypes, brain images, cognitive, and demographic information will be shared with other investigators within Partners (i.e. MGH, BWH) who are not study staff members. We will also make your data available to researchers at other academic institutions, which are not a part of Partners. These are often scientists affiliated with Harvard University working with researchers at MGH and BWH. The coded information will not contain your name or other information that could likely identify you, and investigators will not be given any information that could link the coded information with identifying information.

Your **coded** samples/data, may be shared with for profit companies that are working with Partners researchers. Researchers at for profit companies will never be given your name or other information that could likely identify you. Your samples and/or data will not be sold to anyone for profit.

A coded portion of your DNA sample will be sent to the Broad Institute for whole genome analysis. Usually researchers study a few areas of your genetic code that are linked to a disease or condition. In our whole genome analysis, we will be using all or most of your genes for research related to the brain and psychiatric conditions. The information will be sent with only a code number attached, and your name or other identifiable information will never be given.

In order to allow researchers to share test results, the National Institutes of Health (NIH) and other central repositories have developed special data (information) banks that collect the results of whole genome studies. The NIH or other data store genetic information and give it to other researchers to do more studies. We do not think there will be further risks to your privacy and confidentiality by sharing your whole genome analysis with these databanks; however, we cannot predict predict how genetic information will be used in the future. The information is sent with only a code number attached, and your name or other identifiable information is never given to them. There are safeguards in place to protect your information while it is stored in respositories and used for research.

Research using your whole genome information is important for virtually all disease and conditions. Therefor, the NIH data bank will provide study data for researchers working on any disease, which could include conditions such as Alzheimer's disease, mental illness, cancer, and others.

Subject Identification

May we share your whole genome analysis and genetic information with the NIH and other central repositories?

☐ **Yes** ☐ **No** **Subject Initials** ___________

How long will my DNA samples and information be kept?

There is no scheduled date on which your samples and information in the bank will be destroyed. Your samples may be stored for research until they are "used up."

Can I stop allowing my DNA samples and information to be stored and used for research?

Yes. You have a right to withdraw your permission, at any time and if you do, your samples and your information will be destroyed. However, it will not be possible to destroy samples and information already given to researchers within Partners or at other academic institutions. If you decide to take away your permission, contact

Will I get results of research done using my samples and imaging data?

No. This research is only a stepping stone in understanding the genetic effects on brain function**.** The investigators cannot share genetic or imaging information with you. This information will not be placed in your medical records and will not be useful in directing your medical treatment.

What are the risks and possible discomforts from being in this study?

MRI

There is no extra risk posed by spending an additional 20 to 40 minutes in the scanner. The MRI scan being done is designed to answer research questions, not examine your brain medically.

Genetic Analysis

Saliva collection (i.e. spitting) is generally considered safe and painless. The main risk of allowing us to store and use your samples and certain limited health information for research is a potential loss of privacy. However, we will take the following steps to protect your privacy:

- We will store your samples only with a code in a password protected database.
- The tissue bank database will use the code to connect your sample to certain limited health information about you, but not your name.
- Information that could identify you will only be shared with researchers within Partners who have approval of the Partners ethics board. The board follows state and federal laws and codes of ethics to make sure that the rights and welfare of people taking part in research studies are protected.

- Information that likely could be used to identify you will never be shared with researchers outside Partners.

Genetic information that results from this study does not have medical or treatment importance at this time. However, there is a risk that information about taking part in a genetics study may influence insurance companies and/or employers regarding your health, or have a negative impact on family or other relationships. If you do not share information about taking part in this study with your employer and your medical insurance company, then you will reduce this risk. The study is conducted for research purposes only and we will not place information about the study or the results of study tests in your medical record.
We will do everything we can to keep others from learning about your participation in the research. To further help us protect your privacy, the investigators have obtained a Confidentiality Certificate from the Department of Health and Human Services.

With this Certificate, the investigators cannot be forced (for example by court subpoena) to disclose information that may identify you in any federal, state, or local civil, criminal, administrative, legislative, or other proceedings.

Disclosure will be necessary, however, upon request of DHHS for the purpose of audit or evaluation.

You should understand that a Confidentiality Certificate does not prevent you or a member of your family from voluntarily releasing information about yourself or your involvement in this research. Note however, that if an insurer or employer, learns about your participation, and obtains your consent to receive research information, then the investigator may not use the Certificate of Confidentiality to withhold this information. This means that you and your family must also actively protect your own privacy.

Finally, you should understand that the investigator is not prevented from taking steps, including reporting to authorities, to prevent serious harm to yourself or others.

A Certificate of Confidentiality does not represent an endorsement of this research by the Department of Health and Human Services (DHHS) or the National Institutes of Health (NIH).

Cognitive Testing
There are no known or foreseeable risks or side effects associated with the cognitive tests.

What are the possible benefits from being in this research study?

There are no direct benefits to you for participating. We hope the study results will help us better understand the effects of genes on the brain.

Will I be paid to take part in this research study?

You will be paid $50 for the MRI and DNA collection via a saliva sample. You will receive an additional $60 for completing the online cognitive tasks or $40 for the take-home paper version.

What are the costs to me to take part in this research study?

There will be no cost to you for participating in the study.

Can I still get medical care within Partners if I don't take part in this research tissue bank or if I stop taking part?

Yes. Your decision will not change the medical care you get within Partners now or in the future. There will be no penalty, and you will not lose any benefits you receive now, or have a right to receive. Taking part in the bank is up to you. You can decide not to allow your specimens and information to be placed in the bank. If you decide to take part now, you can change your mind and drop out later.

Whom do I call to answer questions about the bank?

You may ask more questions about the bank at any time. is in charge of this research study. You can call him at with questions.

Whom do I call if I have concerns about my rights as a research subject?

If you want to speak with someone **not** directly involved in the bank project, please contact the ethics board office

You can talk to them about:

- Your rights as a research subject
- Your concerns about the research tissue bank
- A complaint about the research

Also, if you feel pressured to take part in the research tissue bank, or to continue with it, they want to know and can help.

Consent to Collect Samples and Health Information for Research

I confirm that the purpose of the BWH/MGH bank and the potential risks and benefits have been explained to me. All my questions have been answered. I have read this consent form. My

signature below indicates my willingness to have samples and health information about me collected and stored in the bank and used and shared as described above.

Signature of Subject:

______________________________	______________
Adults or Minors, ages 14-17	Date/Time

OR

If you understand the information we have given you, and would like to give your permission for your child/the person you are authorized to represent to take part in this research study, and also agree to allow his/her health information to be used and shared as described above, then please sign below:

Signature of Parent(s)/Guardian or Authorized Representative:

______________________________	______________
Parent(s)/Guardian of Minor	Date/Time

Statement of Study Doctor or Person Obtaining Consent

- I have explained the research to the study subject, and
- I have answered all questions about this research study to the best of my ability.

______________________________	______________
Study Doctor or Person Obtaining Consent	Date/Time
