# Supplementary material for: Brain Genomics Superstruct Project initial data release with structural, functional, and behavioral measures
Source: Sci Data. 2015 Jul 7;2:150031. doi: 10.1038/sdata.2015.31 (PMC4493828; doi:10.1038/sdata.2015.31)
Supplement: Supplementary Appendix B [file sdata201531-s4.pdf]

(0008, 0005)	CharacterSet	CS	10	ISO_IR 100
(0008, 0008)	ImageType	CS	36	ORIGINAL<br>PRIMARY<br>OTHER<br>ND<br>NORM<br>MEAN
(0008, 0012)	InstanceCreationDate	DA	8	OMITTED HERE FOR IDENTIFICATION
(0008, 0013)	InstanceCreationTime	TM	14	OMITTED HERE FOR IDENTIFICATION
(0008, 0016)	SopClass	UI	26	OMITTED HERE FOR IDENTIFICATION
(0008, 0018)	SopInstance	UI	52	OMITTED HERE FOR IDENTIFICATION
(0008, 0020)	StudyDate	DA	8	OMITTED HERE FOR IDENTIFICATION
(0008, 0021)	SeriesDate	DA	8	OMITTED HERE FOR IDENTIFICATION
(0008, 0022)	AcquisitionDate	DA	8	OMITTED HERE FOR IDENTIFICATION
(0008, 0023)	ContentDate	DA	8	OMITTED HERE FOR IDENTIFICATION
(0008, 0030)	StudyTime	TM	14	OMITTED HERE FOR IDENTIFICATION
(0008, 0031)	SeriesTime	TM	14	OMITTED HERE FOR IDENTIFICATION
(0008, 0032)	AcquisitionTime	TM	14	OMITTED HERE FOR IDENTIFICATION
(0008, 0033)	ContentTime	TM	14	OMITTED HERE FOR IDENTIFICATION
(0008, 0050)	AcessionNumber	SH	0	
(0008, 0060)	Modality	CS	2	MR
(0008, 0070)	Manufacturer	LO	8	SIEMENS
(0008, 0080)	InstitutionName	LO	16	OMITTED HERE FOR IDENTIFICATION
(0008, 0081)	InstitutionAddress	ST	32	OMITTED HERE FOR IDENTIFICATION
(0008, 0090)	ReferringPhysician	PN	0	
(0008, 1010)	StationName	SH	6	MEDPC
(0008, 1030)	StudyDescription	LO	22	OMITTED HERE FOR IDENTIFICATION
(0008, 103e)	SeriesDescription	LO	16	T1_MEMPRAGE RMS
(0008, 1050)	PerformingPhysician	PN	0	
(0008, 1070)	OperatorName	PN	6	OMITTED HERE FOR IDENTIFICATION
(0008, 1090)	ModelName	LO	8	TrioTim
(0008, 1140)	ReferencedImageSequence	SQ	306	√æ√ø
(0010, 0010)	PatientName	PN	16	OMITTED HERE FOR IDENTIFICATION
(0010, 0020)	PatientId	LO	48	OMITTED HERE FOR IDENTIFICATION
(0010, 0030)	PatientBirthDate	DA	8	OMITTED HERE FOR IDENTIFICATION
(0010, 0040)	PatientSex	CS	2	F
(0010, 1010)	PatientAge	AS	4	OMITTED HERE FOR IDENTIFICATION
(0010, 1030)	PatientWeight	DS	14	OMITTED HERE FOR IDENTIFICATION
(0018, 0020)	ScanningSequence	CS	6	GR<br>IR
(0018, 0021)	SequenceVariant	CS	6	SP<br>MP
(0018, 0022)	ScanOptions	CS	6	IR<br>PFP
(0018, 0023)	MrAcquisitionType	CS	2	3D
(0018, 0024)	SequenceName	SH	10	tfl3d4_ns
(0018, 0025)	AngioFlag	CS	2	N
(0018, 0050)	SliceThickness	DS	16	1.2000000476837
(0018, 0080)	RepetitionTime	DS	4	2200
(0018, 0081)	EchoTime	DS	4	1.54
(0018, 0082)	InversionTime	DS	4	1100

(0018, 0083)	NumberOfAverages	DS	2	4
(0018, 0084)	ImagingFrequency	DS	10	123.263739
(0018, 0085)	ImagingNucleus	SH	2	1H
(0018, 0086)	EchoNumber	IS	2	1
(0018, 0087)	MagneticFieldStrength	DS	2	3
(0018, 0089)	PhaseEncodingSteps	IS	4	145
(0018, 0091)	EchoTrainLength	IS	2	1
(0018, 0093)	PercentSampling	DS	4	100
(0018, 0094)	PercentPhaseFov	DS	4	100
(0018, 0095)	PixelBandwidth	DS	4	651
(0018, 1000)	DeviceSerialNumber	LO	6	OMITTED HERE FOR IDENTIFICATION
(0018, 1020)	SoftwareVersion	LO	12	syngo MR B17
(0018, 1030)	ProtocolName	LO	12	T1_MEMPRAGE
(0018, 1251)	TransmittingCoil	SH	4	Body
(0018, 1310)	AcquisitionMatrix	US	8	0<br>192<br>192<br>0
(0018, 1312)	PhaseEncodingDirection	CS	4	ROW
(0018, 1314)	FlipAngle	DS	2	7
(0018, 1315)	VariableFlipAngleFlag	CS	2	N
(0018, 1316)	SAR	DS	16	0.07379501370909
(0018, 1318)	DB_DT	DS	2	0
(0018, 5100)	PatientPosition	CS	4	HFS
(0019, 0010)	unknown	LO	18	SIEMENS MR HEADER
(0019, 1008)	unknown	CS	12	IMAGE NUM 4
(0019, 1009)	unknown	LO	4	1.0
(0019, 100b)	unknown	DS	8	131727.5
(0019, 100f)	unknown	SH	4	Fast
(0019, 1011)	unknown	SH	2	No
(0019, 1012)	unknown	SL	12	0<br>0<br>-1275
(0019, 1013)	unknown	SL	12	0<br>0<br>-1275
(0019, 1014)	unknown	IS	6	0<br>0<br>0
(0019, 1015)	unknown	FD	24	-13.5665<br>-123.575<br>105.842
(0019, 1017)	unknown	DS	2	1
(0019, 1018)	unknown	IS	4	4000
(0020, 000d)	StudyInstanceUid	UI	56	1.3.12.2.1107.5.2.32.35380.30000010041618553290100000157
(0020, 000e)	SeriesInstanceUid	UI	58	1.3.12.2.1107.5.2.32.35380.2010042416072655750002973.0.0.0
(0020, 0010)	StudyId	SH	2	1
(0020, 0011)	SeriesNumber	IS	2	5
(0020, 0012)	AcquisitionNumber	IS	2	1
(0020, 0013)	InstanceNumber	IS	2	72
(0020, 0032)	ImagePositionPatient	DS	50	-13.566503167318

				-123.57515817126
				105.84205514206
(0020, 0037)	ImageOrientationPatient	DS	102	0.04179473190323
				0.99780385064773
				-0.0513875083819
				0.03408366365402
				-0.0528263900391
				-0.9980218817177
(0020, 0052)	FrameOfReferenceUid	UI	52	1.3.12.2.1107.5.2.32.35380.1.20100424160346351.0.0.0
(0020, 1040)	PositionReference	LO	0	
(0020, 1041)	SliceLocation	DS	16	-4.7753770674885
(0028, 0002)	SamplesPerPixel	US	2	1
(0028, 0004)	PhotometricInterpretation	CS	12	MONOCHROME2
(0028, 0010)	ImageRows	US	2	192
(0028, 0011)	ImageColumns	US	2	192
(0028, 0030)	PixelSpacing	DS	32	1.1979166269302
				1.1979166269302
(0028, 0100)	BitsAllocated	US	2	16
(0028, 0101)	BitsStored	US	2	12
(0028, 0102)	HighBit	US	2	11
(0028, 0103)	PixelRepresentation	US	2	0
(0028, 0106)	SmallestImagePixelValue	US	2	0
(0028, 0107)	LargestImagePixelValue	US	2	488
(0028, 1050)	WindowCenter	DS	4	281
(0028, 1051)	WindowWidth	DS	4	617
(0028, 1055)	WindowCenterAndWidthExplanation	LO	6	Algo1
(0029, 0010)	unknown	LO	18	SIEMENS CSA HEADER
(0029, 0011)	unknown	LO	22	SIEMENS MEDCOM HEADER2
(0029, 1008)	unknown	CS	12	IMAGE NUM 4
(0029, 1009)	unknown	LO	8	20100424
(0029, 1010)	unknown	OB	9468	

SV10

———————————S

(0029, 1018)	unknown	CS	2	MR
(0029, 1019)	unknown	LO	8	20100424
(0029, 1020)	unknown	OB	58256	

SV10

———————————A

(0029, 1160)	unknown	LO	4	com
(0032, 1060)	RequestedProcedureDescription	LO	22	Investigators Buckner
(0040, 0244)	PerformedProcedureStepStartDate	DA	8	20100424
(0040, 0245)	PerformedProcedureStepStartTime	TM	14	160115.174000
(0040, 0253)	PerformedProcedureStepId	SH	16	MR20100424160115
(0040, 0254)	PerformedProcedureStepDescription	LO	22	Investigators^Buckner
(0051, 0010)	unknown	LO	18	SIEMENS MR HEADER
(0051, 1008)	unknown	CS	12	IMAGE NUM 4
(0051, 1009)	unknown	LO	4	1.0
(0051, 100a)	unknown	LO	8	TA 02:11
(0051, 100b)	unknown	LO	8	192p*192

(0051, 100c)	unknown	LO	12	FoV 229*229
(0051, 100d)	unknown	SH	8	SP R4.8
(0051, 100e)	unknown	LO	22	Sag>Cor(2.3)>Tra(-2.1)
(0051, 100f)	unknown	LO	10	T:HEA;HEP
(0051, 1011)	unknown	LO	2	p4
(0051, 1012)	unknown	SH	4	TP 0
(0051, 1013)	unknown	SH	4	+LPH
(0051, 1016)	unknown	LO	16	p4 ND<br>NORM<br>MEAN
(0051, 1017)	unknown	SH	6	SL 1.2
(0051, 1019)	unknown	LO	10	A4<br>IR<br>PFP
