# Supplementary material for: Brain Genomics Superstruct Project initial data release with structural, functional, and behavioral measures
Source: Sci Data. 2015 Jul 7;2:150031. doi: 10.1038/sdata.2015.31 (PMC4493828; doi:10.1038/sdata.2015.31)
Supplement: Supplementary Appendix C [file sdata201531-s5.pdf]

(0008, 0005)	CharacterSet	CS	10	ISO_IR 100
(0008, 0008)	ImageType	CS	28	ORIGINAL
				PRIMARY
				M
				ND
				MOSAIC
(0008, 0012)	InstanceCreationDate	DA	8	OMITTED HERE FOR IDENTIFICATION
(0008, 0013)	InstanceCreationTime	TM	14	OMITTED HERE FOR IDENTIFICATION
(0008, 0016)	SopClass	UI	26	OMITTED HERE FOR IDENTIFICATION
(0008, 0018)	SopInstance	UI	52	OMITTED HERE FOR IDENTIFICATION
(0008, 0020)	StudyDate	DA	8	OMITTED HERE FOR IDENTIFICATION
(0008, 0021)	SeriesDate	DA	8	OMITTED HERE FOR IDENTIFICATION
(0008, 0022)	AcquisitionDate	DA	8	OMITTED HERE FOR IDENTIFICATION
(0008, 0023)	ContentDate	DA	8	OMITTED HERE FOR IDENTIFICATION
(0008, 0030)	StudyTime	TM	14	OMITTED HERE FOR IDENTIFICATION
(0008, 0031)	SeriesTime	TM	14	OMITTED HERE FOR IDENTIFICATION
(0008, 0032)	AcquisitionTime	TM	14	OMITTED HERE FOR IDENTIFICATION
(0008, 0033)	ContentTime	TM	14	OMITTED HERE FOR IDENTIFICATION
(0008, 0050)	AcessionNumber	SH	0	
(0008, 0060)	Modality	CS	2	MR
(0008, 0070)	Manufacturer	LO	8	SIEMENS
(0008, 0080)	InstitutionName	LO	16	OMITTED HERE FOR IDENTIFICATION
(0008, 0081)	InstitutionAddress	ST	32	OMITTED HERE FOR IDENTIFICATION
(0008, 0090)	ReferringPhysician	PN	0	
(0008, 1010)	StationName	SH	6	MEDPC
(0008, 1030)	StudyDescription	LO	22	OMITTED HERE FOR IDENTIFICATION
(0008, 103e)	SeriesDescription	LO	18	fMRI_resting_state
(0008, 1050)	PerformingPhysician	PN	0	
(0008, 1070)	OperatorName	PN	6	OMITTED HERE FOR IDENTIFICATION
(0008, 1090)	ModelName	LO	8	TrioTim
(0008, 1140)	ReferencedImageSequence	SQ	306	√æ√ø
(0010, 0010)	PatientName	PN	16	OMITTED HERE FOR IDENTIFICATION
(0010, 0020)	PatientId	LO	48	OMITTED HERE FOR IDENTIFICATION
(0010, 0030)	PatientBirthDate	DA	8	OMITTED HERE FOR IDENTIFICATION
(0010, 0040)	PatientSex	CS	2	F
(0010, 1010)	PatientAge	AS	4	OMITTED HERE FOR IDENTIFICATION
(0010, 1030)	PatientWeight	DS	14	OMITTED HERE FOR IDENTIFICATION
(0018, 0020)	ScanningSequence	CS	2	EP
(0018, 0021)	SequenceVariant	CS	2	SK
(0018, 0022)	ScanOptions	CS	2	FS
(0018, 0023)	MrAcquisitionType	CS	2	2D
(0018, 0024)	SequenceName	SH	12	epfid2d1_72
(0018, 0025)	AngioFlag	CS	2	N
(0018, 0050)	SliceThickness	DS	2	3
(0018, 0080)	RepetitionTime	DS	4	3000
(0018, 0081)	EchoTime	DS	2	30
(0018, 0083)	NumberOfAverages	DS	2	1
(0018, 0084)	ImagingFrequency	DS	10	123.26374
(0018, 0085)	ImagingNucleus	SH	2	1H
(0018, 0086)	EchoNumber	IS	2	1
(0018, 0087)	MagneticFieldStrength	DS	2	3

(0018, 0088)	SliceSpacing	DS	16	2.9999999039362
(0018, 0089)	PhaseEncodingSteps	IS	2	72
(0018, 0091)	EchoTrainLength	IS	2	1
(0018, 0093)	PercentSampling	DS	4	100
(0018, 0094)	PercentPhaseFov	DS	4	100
(0018, 0095)	PixelBandwidth	DS	4	2240
(0018, 1000)	DeviceSerialNumber	LO	6	OMITTED HERE FOR IDENTIFICATION
(0018, 1020)	SoftwareVersion	LO	12	syngo MR B17
(0018, 1030)	ProtocolName	LO	18	fMRI_resting_state
(0018, 1251)	TransmittingCoil	SH	4	Body
(0018, 1310)	AcquisitionMatrix	US	8	72
				0
				0
				72
(0018, 1312)	PhaseEncodingDirection	CS	4	COL
(0018, 1314)	FlipAngle	DS	2	85
(0018, 1315)	VariableFlipAngleFlag	CS	2	N
(0018, 1316)	SAR	DS	16	0.14298183329098
(0018, 1318)	DB_DT	DS	2	0
(0018, 5100)	PatientPosition	CS	4	HFS
(0019, 0010)	unknown	LO	18	SIEMENS MR HEADER
(0019, 1008)	unknown	CS	12	IMAGE NUM 4
(0019, 1009)	unknown	LO	4	1.0
(0019, 100a)	unknown	US	2	47
(0019, 100b)	unknown	DS	2	35
(0019, 100f)	unknown	SH	4	Fast
(0019, 1011)	unknown	SH	2	No
(0019, 1012)	unknown	SL	12	0
				0
				-1275
(0019, 1013)	unknown	SL	12	0
				0
				-1275
(0019, 1014)	unknown	IS	6	0
				0
				0
(0019, 1015)	unknown	FD	24	-788.847
				-736.79
				-52.9814
(0019, 1016)	unknown	DS	6	96.385
(0019, 1017)	unknown	DS	2	1
(0019, 1018)	unknown	IS	4	3100
(0019, 1028)	unknown	FD	8	27.233
(0019, 1029)	unknown	FD	376	0
				1537.5
				65
				1602.5
				127.5
				1665
				192.5
				1730

```
                                                                          255
                                                                          1795
                                                                          320
                                                                          1857.5
                                                                          385
                                                                          1922.5
                                                                          447.5
                                                                          1985
                                                                          512.5
                                                                          2050
                                                                          577.5
                                                                          2115
                                                                          640
                                                                          2177.5
                                                                          705
                                                                          2242.5
                                                                          770
                                                                          2307.5
                                                                          832.5
                                                                          2370
                                                                          897.5
                                                                          2435
                                                                          960
                                                                          2500
                                                                          1025
                                                                          2562.5
                                                                          1090
                                                                          2627.5
                                                                          1152.5
                                                                          2690
                                                                          1217.5
                                                                          2755
                                                                          1282.5
                                                                          2820
                                                                          1345
                                                                          2882.5
                                                                          1410
                                                                          2947.5
                                                                          1472.5
(0020, 000d)  StudyInstanceUid                   UI            56 OMITTED HERE FOR IDENTIFICATION
(0020, 000e)  SeriesInstanceUid                  UI            58 OMITTED HERE FOR IDENTIFICATION
(0020, 0010)  StudyId                            SH             2  1
(0020, 0011)  SeriesNumber                       IS             2  14
(0020, 0012)  AcquisitionNumber                  IS             2  33
(0020, 0013)  InstanceNumber                     IS             2  33
(0020, 0032)  ImagePositionPatient               DS            50  -788.84719829265
                                                                   -736.78994392663
                                                                   -52.981431394183
(0020, 0037)  ImageOrientationPatient            DS           100  0.9985446782004
                                                                   -0.0399606693677
                                                                   0.03621699243911
```

```
                                                                    0.04179484065056
                                                                    0.99780383837675
                                                                    -0.0513876582033
(0020, 0052)  FrameOfReferenceUid                  UI           52  1.3.12.2.1107.5.2.32.35380.1.20100424160346351.0.0.0
(0020, 1040)  PositionReference                    LO            0
(0020, 1041)  SliceLocation                        DS           16  -64.911658774742
(0028, 0002)  SamplesPerPixel                      US            2  1
(0028, 0004)  PhotometricInterpretation            CS           12  MONOCHROME2
(0028, 0010)  ImageRows                            US            2  504
(0028, 0011)  ImageColumns                         US            2  504
(0028, 0030)  PixelSpacing                         DS            4  3
                                                                    3
(0028, 0100)  BitsAllocated                        US            2  16
(0028, 0101)  BitsStored                           US            2  12
(0028, 0102)  HighBit                              US            2  11
(0028, 0103)  PixelRepresentation                  US            2  0
(0028, 0106)  SmallestImagePixelValue              US            2  0
(0028, 0107)  LargestImagePixelValue               US            2  1887
(0028, 1050)  WindowCenter                         DS            4  703
(0028, 1051)  WindowWidth                          DS            4  1517
(0028, 1055)  WindowCenterAndWidthExplanation      LO            6  Algo1
(0029, 0010)  unknown                              LO           18  SIEMENS CSA HEADER
(0029, 0011)  unknown                              LO           22  SIEMENS MEDCOM HEADER2
(0029, 1008)  unknown                              CS           12  IMAGE NUM 4
(0029, 1009)  unknown                              LO            8  20100424
(0029, 1010)  unknown                              OB        11220
SV10
________________________________________________________________________________________________________
__________________________S
(0029, 1018)  unknown                              CS            2  MR
(0029, 1019)  unknown                              LO            8  20100424
(0029, 1020)  unknown                              OB        86208
SV10
________________________________________________________________________________________________________
__________________________A
(0029, 1160)  unknown                              LO            4  com
(0032, 1060)  RequestedProcedureDescription        LO           22 OMITTED HERE FOR IDENTIFICATION
(0040, 0244)  PerformedProcedureStepStartDate      DA            8 OMITTED HERE FOR IDENTIFICATION
(0040, 0245)  PerformedProcedureStepStartTime      TM           14 OMITTED HERE FOR IDENTIFICATION
(0040, 0253)  PerformedProcedureStepId             SH           16 OMITTED HERE FOR IDENTIFICATION
(0040, 0254)  PerformedProcedureStepDescription    LO           22 OMITTED HERE FOR IDENTIFICATION
(0051, 0010)  unknown                              LO           18  SIEMENS MR HEADER
(0051, 1008)  unknown                              CS           12  IMAGE NUM 4
(0051, 1009)  unknown                              LO            4  1.0
(0051, 100a)  unknown                              LO            8  TA 00.03
(0051, 100b)  unknown                              LO            6  72*72
(0051, 100c)  unknown                              LO           14  FoV 1512*1512
(0051, 100e)  unknown                              LO           22  Tra>Cor(-3.0)>Sag(2.0)
(0051, 100f)  unknown                              LO           10  C:HEA;HEP
(0051, 1012)  unknown                              SH            4  TP 0
(0051, 1013)  unknown                              SH            4  +LPH
```

(0051, 1016)	unknown	LO	12	M<br>ND<br>MOSAIC
(0051, 1017)	unknown	SH	6	SL 3.0
(0051, 1019)	unknown	LO	6	A1<br>FS
