# Supplementary material for: Brain Genomics Superstruct Project initial data release with structural, functional, and behavioral measures
Source: Sci Data. 2015 Jul 7;2:150031. doi: 10.1038/sdata.2015.31 (PMC4493828; doi:10.1038/sdata.2015.31)
Supplement: Supplementary Appendix D [file sdata201531-s6.pdf]

Phenotypes Legend

Phenotype	Description
Subject_ID	The anonymous data release ID.
Delay	The number of days between test and retest scans.
Subject_Rescan_ID	The anonymous data release ID for subjects who were re-scanned within six months of their initial scan date.
MRI	Reflects the number of eyes open rest runs available (1, 2).
Behavior	Reflects the presence of behavioral data (Present, Absent).
Sex	Sex (M, F).
Age_Bin	The binned age (2 year bins) of each participant at the time of image acquisition. Participants who were from 18-19 years of age at the point of scan are coded as 19, participants who were 20-21 years of age are coded as 21, etc.
Hand	Participant handedness (RHT, LFT, AMB).
Educ	Years of education. Please note, due to the characteristics of the sample education is truncated by the age of the participant and should not be interpreted as an accurate reflection (nor a proxy) of SES.
Race_Ethn	Participant race/ethnicity (White not Hispanic = W_NOT_HL, all other race/ethnicities = Other).
Scanner_Bin	Site/Scanner bay where the data were acquired (A, B, C, D, E).
Console	Console software version on scanner at the time of image acquisition (B13, B15, B17).
Coil	Coil version (currently all 12-channel coils = Tim_12).
ANAT	The run number for the T1 MEMPRAGE anatomical scan (1).
BOLD1	The run number for the first eyes open rest run (2).
BOLD1_sSNR	Slice based SNR for first eyes open rest run.
BOLD1_MotMicro	Number of relative translations in 3D space >= 0.1mm.
BOLD1_MotAbsMax	Maximum absolute translation in 3D space (mm).
BOLD2	The run number for the second eyes open rest run when present (3).

BOLD2_sSNR	Slice based SNR for second eyes open rest run.
BOLD2_MotMicro	Number of relative translations in 3D space >= 0.1mm.
BOLD2_MotAbsMax	Maximum absolute translation in 3D space (mm).
Flank_S_CORRpc	The percentage of correct responses for the flanker task during switch blocks.
Flank_S_meanRTcorr	The mean reaction time (RT) for correct flanker task trials during switch blocks.
Flank_S_medRTcorr	The median RT for correct flanker task trials during switch blocks.
Flank_S_score	The number of correct trials minus the number of incorrect trials for the switch blocks.
Flank_NS_CORRpc	The percentage of correct responses for the flanker task during non-switch blocks.
Flank_NS_meanRTcorr	The mean RT for the correct flanker task trials during non-switch blocks.
Flank_NS_medRTcorr	The median RT for the correct flanker task trials during the non-switch blocks.
Flank_NS_score	The number of correct trials minus the number of incorrect trials for the non-switch blocks.
Flank_CORRpc	The percent of correct responses for the flanker task.
Flank_meanRTcorr	The mean flanker task RT for correct responses.
Flank_medRTcorr	The median flanker task RT for correct responses.
Flank_TOT_score	The number of correct trials minus the number of incorrect trials for the flanker task.
MenRot_0_CORRpc	Percent of correct responses for mental rotation 0-degree rotation trials.
MenRot_0_meanRTcorr	Mean RT of correct responses for mental rotation 0-degree rotation trials.
MenRot_0_medRTcorr	Median RT of correct responses for mental rotation 0-degree rotation trials.
MenRot_80_CORRpc	Percent of correct responses for mental rotation 80-degree rotation trials.

MenRot_80_meanRTcorr	Mean RT of correct responses for mental rotation 80-degree rotation trials.
MenRot_80_medRTcorr	Median RT of correct responses for mental rotation 80-degree rotation trials.
MenRot_120_CORRpc	Percent of correct responses for mental rotation 120-degree rotation trials.
MenRot_120_meanRTcorr	Mean RT of correct responses for mental rotation 120-degree rotation trials.
MenRot_120_medRTcorr	Median RT of correct responses for mental rotation 120-degree rotation trials.
MenRot_160_CORRpc	Mental rotation percent of correct responses for 160-degree rotation trials.
MenRot_160_meanRTcorr	Mean RT of correct responses for mental rotation 160-degree rotation trials.
MenRot_160_medRTcorr	Median RT of correct responses for mental rotation 160-degree rotation trials.
MenRot_TOT_CORRpc	Percent of correct responses for mental rotation task.
MenRot_TOT_meanRTcorr	Mean RT of correct responses for mental rotation task.
MenRot_TOT_medRTcorr	Median RT of correct responses for mental rotation task.
ICV	Estimated total intracranial volume (mm^3; Buckner et al., 2004).
BrainSegVol	The volume of brain as the sum of the volumes of the segmentations that are in the brain.
BrainSegVolNonVent	The volume of brain as the sum of the volumes of the segmentations that are in the brain excluding the ventricles.
postCorCall_Vol	Posterior corpus callosum (mm^3).
midpostCorCall_Vol	Middle posterior corpus callosum (mm^3).
centCorCall_Vol	Central corpus callosum (mm^3).
midantCorCall_Vol	Middle anterior corpus callosum (mm^3).
antCorCall_Vol	Anterior corpus callosum (mm^3).
R_AvgCortThick	Right hemisphere average cortical thickness (mm).
L_AvgCortThick	Left hemisphere average cortical thickness (mm).

R_TotCortSurfArea	Right hemisphere total cortical surface area (mm^2).
L_TotCortSurfArea	Left hemisphere total cortical surface area (mm^2).
R_Amy_Vol	Right hemisphere amygdala volume (mm^3).
L_Amy_Vol	Left hemisphere amygdala volume (mm^3).
R_Hipp_Vol	Right hemisphere hippocampal volume (mm^3).
L_Hipp_Vol	Left hemisphere hippocampal volume (mm^3).
R_rACC_Thick	Right hemisphere rostral anterior cingulate cortical thickness (mm).
L_rACC_Thick	Left hemisphere rostral anterior cingulate cortical thickness (mm).
R_cMF_Thick	Right hemisphere caudal middle frontal cortical thickness (mm).
L_cMF_Thick	Left hemisphere caudal middle frontal cortical thickness (mm).
R_lOcc_Thick	Right hemisphere lateral occipital thickness (mm).
L_lOcc_Thick	Left hemisphere lateral occipital thickness (mm).
R_lingual_Thick	Right hemisphere lingual thickness (mm).
L_lingual_Thick	Left hemisphere lingual thickness (mm).
R_cACC_Thick	Right caudal anterior cingulate thickness (mm).
L_cACC_Thick	Left caudal anterior cingulate thickness (mm).
R_PCC_Thick	Right posterior cingulate thickness (mm).
L_PCC_Thick	Left posterior cingulate thickness (mm).
R_isthmusACC_Thick	Right isthmus cingulate thickness (mm).
L_isthmusACC_Thick	Left isthmus cingulate thickness (mm).
R_Parahipp_Thick	Right parahippocampal thickness (mm).
L_Parahipp_Thick	Left parahippocampal thickness (mm).
R_Fform_Thick	Right fusiform thickness (mm).
L_Fform_Thick	Left fusiform thickness (mm).
R_supF_Thick	Right superiorfrontal thickness (mm).

L_supF_Thick	Left superiorfrontal thickness (mm).
R_iPar_Thick	Right inferiorparietal thickness (mm).
L_iPar_Thick	Left inferiorparietal thickness (mm).
R_Ins_Thick	Right insula thickness (mm).
L_Ins_Thick	Left insula thickness (mm).
Health_Rating	*Compared to other people how would you rate your physical health? (1 – much worse than average; 2 – worse than average; 3 – average; 4 – better than average; 5 – much better than average).*
Health_Satisfy	*How satisfied are you with your present health? (1 – not at all satisfied; 2 – not very satisfied; 3 – neither satisfied nor dissatisfies; 4 – somewhat satisfied; 5 – extremely satisfied).*
STAI_tAnxiety	*State-trait anxiety inventory for adults; Measure of trait anxiety (Score range 20-80; Spielberger and Gorsuch, 1970).*
STAI_sAnxiety	*State-trait anxiety inventory for adults; Measure of state anxiety (Score range 20-80).*
NEO_N	*The NEO Five-factor model of personality; Neuroticism score (Score range 0-48; Costa and McCrae, 1992).*
NEO_E	*The NEO Five-factor model of personality; Extraversion score (Score range 0-48).*
NEO_O	*The NEO Five-factor model of personality; Openness score (Score range 0-48).*
NEO_A	*The NEO Five-factor model of personality; Agreeableness score (Score range 0-48).*
NEO_C	*The NEO Five-factor model of personality; Conscientiousness score (Score range 0-48).*
BISBAS_BAS_Drive	*Behavioral inhibition (BIS) and behavioral activation (BAS) scale; BAS drive score (Score range 4-16; Carver and White, 1994).*
BISBAS_BAS_Fun	*Behavioral inhibition (BIS) and behavioral activation (BAS) scale; BAS funseeking score (Score range 4-16).*
BISBAS_BAS_Reward	*Behavioral inhibition (BIS) and behavioral activation (BAS) scale; BAS reward score (Score range 5-20).*
BISBAS_BIS	*Behavioral inhibition (BIS) and behavioral activation (BAS) scale; BIS score (Score range 7-28).*

MindWandering_Freq	*Imaginal process inventory; 12-question mind wandering subscale (Score range 12-60; Singer and Antrobus, 1970)*
Barratt_tot	*Barratt Impulsivity Scale; Total score (Score range 30-120; Patton et al., 1995).*
Barratt_2atten	*Barratt Impulsivity Scale; 2nd order attentional impulsiveness factor (Score range 8-32).*
Barratt_2mot	*Barratt Impulsivity Scale; 2nd order motor factor (Score range 11-44).*
Barratt_2nonplan	*Barratt Impulsivity Scale; 2nd order non-planning factor (Score range 11-44).*
Barratt_1atten	*Barratt Impulsivity Scale; 1st order attentional factor (Score range 5-20).*
Barratt_1mot	*Barratt Impulsivity Scale; 1st order motor factor (Score range 7-28).*
Barratt_1selfcontrol	*Barratt Impulsivity Scale; 1st order self-control factor (Score range 6-24).*
Barratt_1complex	*Barratt Impulsivity Scale; 1st order cognitive complexity factor (Score range 5-20).*
Barratt_1persever	*Barratt Impulsivity Scale; 1st order perseverance factor (Score range 4-16).*
Barratt_1instability	*Barratt Impulsivity Scale; 1st order cognitive instability factor (Score range 3-12).*
DOSPERT_taking	*Domain-specific risk-tasking scale; Risk taking (Score range 40-280; Weber et al., 2002).*
DOSPERT_perception	*Domain-specific risk-tasking scale; Risk perception (Score range 40-280).*
POMS_TotMdDisturb	*Profile of Mood States; Total Mood Disturbance score (Score range -20-100; McNair et al., 1971).*
POMS_T_TensionAnxiety	*Profile of Mood States; T-score Tension/Anxiety (Score range 30-67).*
POMS_T_DepressionDejection	*Profile of Mood States; T-score Depression/Dejection (Score range 32-69).*
POMS_T_AngerHostility	*Profile of Mood States; T-score Anger/Hostility (Score range 36-76).*
POMS_T_VigorActivity	*Profile of Mood States; T-score Vigor/Activity (Score range 36-80).*
POMS_T_FatigueInertia	*Profile of Mood States; T-score Fatigue/Inertia (Score range 30-76).*

POMS_T_ConfusionBewilderment	*Profile of Mood States; T-score Confusion/Bewilderment (Score range 33-75).*
TCI_Novelty	*Temperament and Character Inventory (TCI-9); Novelty-seeking (Score range 20-100; Cloninger, 1987).*
TCI_RewardDependence	*Temperament and Character Inventory (TCI-9); Reward Dependence (Score range 20-100).*
TCI_HarmAvoidance	*Temperament and Character Inventory (TCI-9); Harm Avoidance (Score range 20-100).*
Shipley_Vocab_Raw	*Raw number correct for the Shipley vocabulary task (Score range 0-40).*
EstIQ_Shipley_Int_Bin	*Estimated IQ derived from Shipley-Hartford Age-Corrected T-Scores. Reported values are in integers. IQ scores are binned (2-point bins). Participants who scored from 124-125 are coded as 125, participants who scored from from 98-99 are coded as 99, etc.*
Matrix_WAIS	*Matrix reasoning Wechsler Adult Intelligence Scale (WAIS) score.*<br><br>*Scoring rules are as follows:*<br>*1. Examinee receives 1 point for each correct response.*<br>*2. If the examinee obtains perfect scores on items 4 and 5, give full credit for items 1-3.*<br>*3. Discontinue after 4 consecutive errors or 4 errors on five consecutive trials.*<br>*4. Count trials with RT < 300ms as errors.*
EstIQ_Matrix_Int_Bin	*Estimated IQ derived through the OPIE3 formula (Schoenberg et al., 2002). Reported values are in integers. IQ scores are binned (2-point bins). Participants who scored from 124-125 are coded as 125, participants who scored from from 98-99 are coded as 99, etc.*

Note: Phenotypes listed in italics are available when requesting the extended data release (http://neuroinformatics.harvard.edu/gsp/get/).
